# Expanding the Occurrence of Polysaccharides to the Viral World: The Case of Mimivirus

**DOI:** 10.1002/anie.202106671

**Published:** 2021-08-03

**Authors:** Anna Notaro, Yohann Couté, Lucid Belmudes, Maria Elena Laugieri, Annalisa Salis, Gianluca Damonte, Antonio Molinaro, Michela G. Tonetti, Chantal Abergel, Cristina De Castro

**Affiliations:** ^1^ Department of Chemical Sciences University of Naples Federico II Via Cinthia 21 80126 Naples Italy; ^2^ Information Génomique & Structurale Unité Mixte de Recherche 7256 Aix-Marseille University Centre National de la Recherche Scientifique, IMM, IM2B 13288 Marseille Cedex 9 France; ^3^ INSERM, CEA, UMR BioSanté U1292 Univ. Grenoble Alpes CNRS, CEA, FR2048 38000 Grenoble France; ^4^ Department of Experimental Medicine and Center of Excellence for Biomedical Research University of Genova Genova Italy; ^5^ Department of Agricultural Sciences University of Naples Federico II Via Università, 100 80055 Portici (NA) Italy

**Keywords:** giant viruses, glycans, glycocalyx, Mimivirus, NMR spectroscopy

## Abstract

The general perception of viruses is that they are small in terms of size and genome, and that they hijack the host machinery to glycosylate their capsid. Giant viruses subvert all these concepts: their particles are not small, and their genome is more complex than that of some bacteria. Regarding glycosylation, this concept has been already challenged by the finding that Chloroviruses have an autonomous glycosylation machinery that produces oligosaccharides similar in size to those of small viruses (6–12 units), albeit different in structure compared to the viral counterparts. We report herein that Mimivirus possesses a glycocalyx made of two different polysaccharides, now challenging the concept that all viruses coat their capsids with oligosaccharides of discrete size. This discovery contradicts the paradigm that such macromolecules are absent in viruses, blurring the boundaries between giant viruses and the cellular world and opening new avenues in the field of viral glycobiology.

## Graphical abstract

The giant virus Mimivirus has an icosahedral capsid embedded in a thick layer of fibrils, resembling the glycocalyx of many bacteria. This study revealed that these fibrils are composed by a limited set of carrier proteins which bear two distinct polysaccharides. The presence of polysaccharides refutes the paradigm that such macromolecules are absent in viruses and opens new avenues in the field of glycosciences and viral biology.

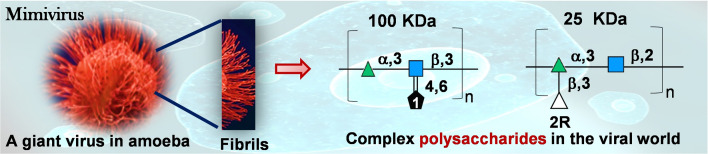

## Introduction

Glycans, either alone or linked to a carrier, are molecules essential in all the domains of life, and the way they occur has been continuously challenged. For instance, N‐linked glycans were initially thought to be restricted to Eukarya, but now it is clear that they are produced by both Bacteria and Archaea, using biosynthetic pathways which share many analogies among all of them, in spite of the different monosaccharide architecture.^[1]^ Similarly, O‐linked glycoproteins have been found to be ubiquitous too, from the small mucins or the huge proteoglycans in animals, to the unusual glycans that decorate the flagellins in Bacteria and the S‐layer proteins in Archaea.

Concerning viruses, the general view is that they do not encode any of the proteins needed for the glycosylation process. Nevertheless, many viruses, like Ebola,^[2]^ or HIV,^[3]^ decorate their capsid proteins with oligosaccharides of discrete size (6–12 units) entirely synthetized and attached by the host glycosylation machinery. Accordingly, the glycans of these viruses resemble those of the host.

Moreover, this general view on viruses has been challenged by the study of the giant virus *Paramecium bursaria Chlorella* virus (PBCV‐1). In contrast with the Ebola virus and HIV, PBCV‐1 encodes a complex set of glyco‐related genes, as those involved in sugar‐nucleotide metabolism, as well as glycosyltransferases,^[4]^ and its major capsid protein is N‐glycosylated in atypical sequons of the protein. The oligosaccharides are about 8–10 sugars in size,^[5]^ and their structure has no equivalent in any domain of life. These same features are shared by other Chloroviruses,[^6^, ^7^, ^8^] and taken all together this supports the hypothesis that these giant viruses encode most, if not all, the enzymes necessary to glycosylate their capsid proteins.

It is worth mentioning that, independently from the mechanism used by any virus to glycosylate their capsid, the size of the glycans has been always restricted to a handful of monosaccharide residues.

Mimivirus is the prototype of the growing family of *Acanthamoeba* infecting *Mimiviridae*,^[9]^ and it belongs to a genus divided in four different clades: *Mimiviruses*, *Megaviruses*, *Moumouviruses* and the most distant *Tupanviruses*, all replicating in the amoebozoan genus *Acanthamoeba* cytoplasm. Since its discovery,^[9b]^ Mimivirus disrupted many long lasting beliefs, with its 1000 genes outnumbering those found in all the viruses known at the time of its discovery.[^9a^, ^10^]

**Figure 1:**
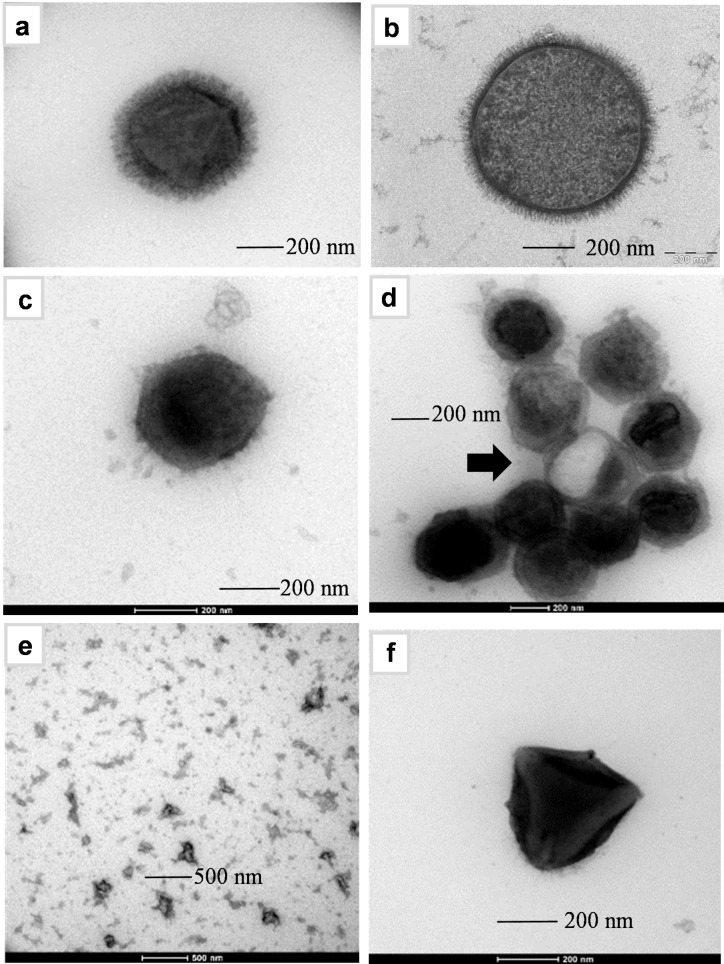
Transmission electron microscopy images of a) control, Mimivirus untreated; b) Bacillus subtilis;^[11]^ c) viral particle after treatment with DTT 50 mM, 2 h, at 100 °C; d) as in (c), about 1/10 of the viral particles appeared damaged (black arrow); e) fibrils released from the treatment and appearing as aggregates; f) M4, a Mimivirus isolated in laboratory condition, which is lacking fibrils like the virus treated with DTT in (d).

From a morphological viewpoint, Mimivirus 450 nm icosahedral capsid is covered with a thick layer of fibrils of about ≈120 nm in length (Figure 1 a), that reminds the glycocalyx of many bacteria, as that of *Bacillus subtilis* reported for comparison (Figure 1 b). This observation prompted the hypothesis that the fibrils could be heavily glycosylated[^9a^, ^12^] in accordance with the presence of many genes involved in glycan formation, some of which having been characterized at the molecular level as carbohydrate active enzymes (CAZymes) involved in the synthesis of monosaccharides such as L‐rhamnose (Rha),^[13]^ N‐acetyl‐glucosamine (GlcNAc),^[14]^ and the rare 2‐O‐methyl‐4 N‐acetyl‐viosamine (2OMeVio4NAc).^[15]^

Moreover, the intricate network appearance of the fibrils layer also suggested the capsid could be glycosylated in a different way compared to other viruses, namely not with oligosaccharides of discrete size, but with glycans in the range of several thousands of Daltons.

Based on this premise, the structural study of the fibrils was undertaken to correlate the phenotype of the fibrils to their chemical nature.

## Results and Discussion

### Release and Chemical Analysis of Fibrils

The isolation of the fibrils from the virus, was possible by adapting the conditions previously described.^[16]^ In our hands, the best condition relied on heating at 100 °C the viral particles in the presence of DTT (Figures 1 c–e) for 2 h. This treatment returned bald virions (Figures 1 c,d), with only few damaged (less than 10 % on average, Figure 1 d) while the fibrils were found in the medium as aggregates due to heating (Figure 1 e). Then, monosaccharide composition of the fibrils as acetylated *O*‐methyl glycosides, confirmed the presence of Rha, 2OMeVio4NAc, and GlcNAc, along with minor amounts of non‐methylated Vio4NAc, mannose and glucose (Figure S1a), while linkage analysis revealed 3‐substituted rhamnose (3‐Rha), 2,3‐Rha, 3‐GlcN, 3,4,6‐GlcN, and terminal‐Vio4NAc (Figure S2) that derived from both Vio4NAc and 2OMeVioNAc units. Conversely, the analysis of the defibered viral particles (Figures 1 c,d) identified some glucose and glucosamine at level of the instrumental baseline noise, demonstrating the location of the sugars primarily on the fibrils. Similar results were obtained by analyzing Mimivirus M4 particles (Figure S1a), a fibril‐deficient mutant of Mimivirus (Figure 1 f).^[17]^ M4 isolate is the result of a 150 times subculture of Mimivirus in a germ‐free amoebal host, that led to dramatic genome reduction from 1.20 Mbp to 0.993 Mbp due to two large deletions, mainly at the two extremities of the genome. One of these large deletions includes the genes encoding for the proteins known to be involved in the biosynthesis of the sugars,[^13^, ^15^] as well as some structural proteins composing the fibrils.^[18]^

In agreement with the information above and our hypothesis, M4 showed no monosaccharide residues, strengthening the evidence that the glycans were attached to Mimivirus fibrils.

### NMR Analysis of the Intact Fibrils of Mimivirus

**Figure 2:**
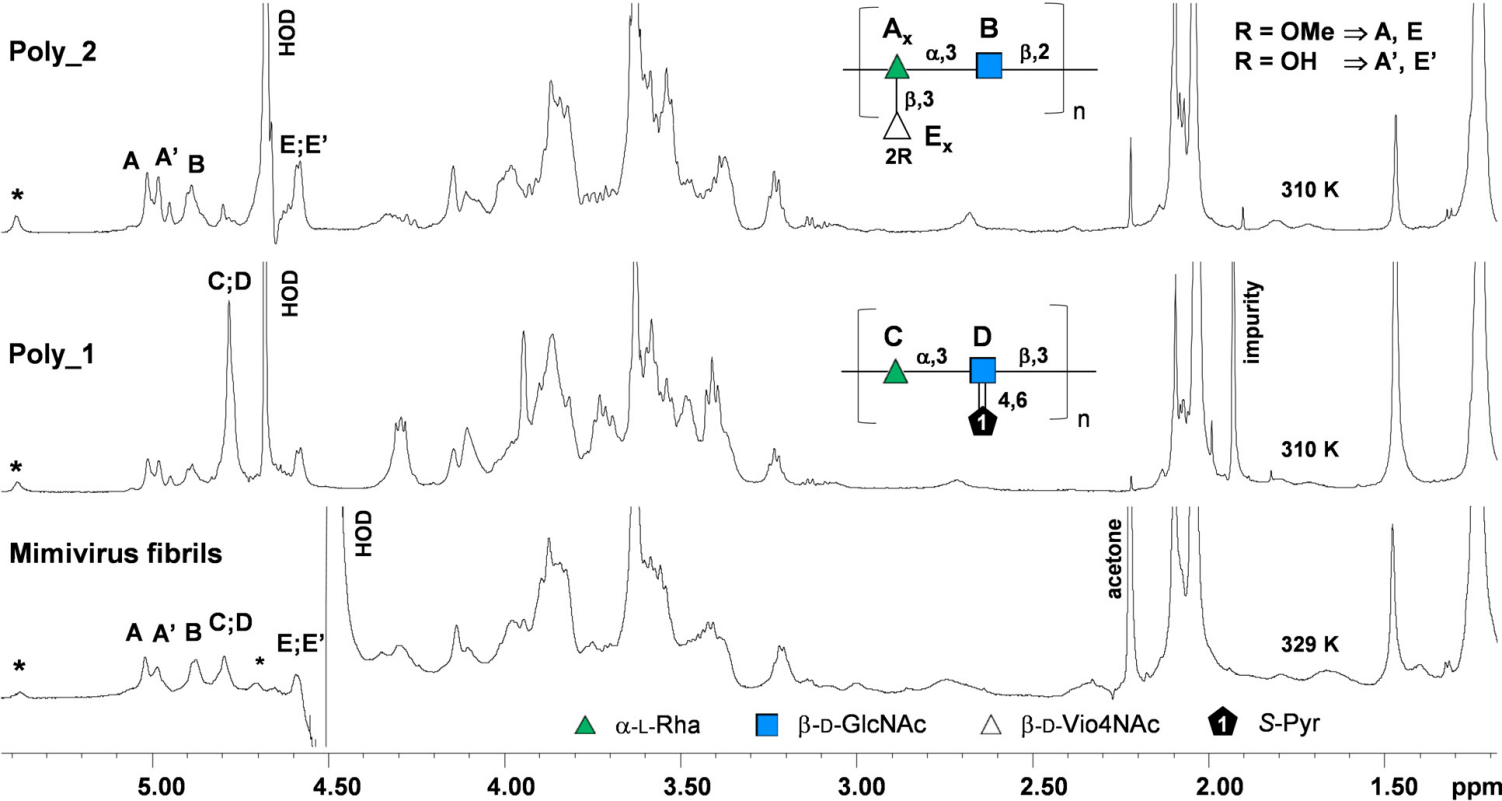
NMR analysis of the fibrils. Proton spectra measured at 600 MHz (the temperature is indicated on each spectrum) of the intact fibrils and of the glycans purified by anion exchange chromatography after two protease digestions, poly_1 (from 400 mM fraction) and poly_2 (from 100 mM fraction), along with their structures. Letters used for the annotation of the signals follow the system of Table S1, and are reported next to the corresponding residue in the structure of the repeating unit. As for poly_2, viosamine is labelled **E** when it is methylated at O‐2 (R=OMe), and the linked rhamnose unit is labelled **A**; when the methyl is absent (R=OH) viosamine and rhamnose are labelled **E′** and **A′**, respectively. * indicates minor anomeric signals whose identity could not be established.

In order to elucidate the nature of the resulting glycans, NMR analysis was carried out on the intact fibrils. The ^1^H NMR spectrum (Figure 2) displayed several signals of different intensities in the region typical for the anomeric signals of the glycans (5.5–4.4 ppm).

Beside the anomeric region, there was a crowded ring proton signals area (4.3–3.2 ppm), and several types of methyl groups: one O‐linked (3.63 ppm), some N‐acetyl groups (2.09–2.07 ppm), one pyruvate (1.47 ppm), and an intense signal at 1.24 ppm related to the methyl group of several 6‐deoxy‐sugars (Figure 2).

The HSQC spectrum (Figure S3a) disclosed the presence of proteins as denoted from the diagnostic ^13^C signals at ≈55 and ≈43 ppm, typical of the C_α_ of amino acids.

Finally, TOCSY (Figures S4b and S4c) and HSQC (Figure S3) spectra indicated that some of the signals in the anomeric region resulted from the overlap of the anomeric protons of more than one monosaccharide unit, as for instance those at 5.0 and 4.8 ppm.

The assignment of the full set of 2D NMR spectra (Table S1) was achieved for the most intense signals, seven in total, each labeled with a capital letter (Figures S3 and S4). The analysis focused on the most intense signals related to carbohydrates (chemical shifts in Table S1). Residue **A** (5.02 ppm) was an α‐Rha, based on the diagnostic pattern found in the COSY and TOCSY spectra (Figure S4b). Indeed, the TOCSY spectrum displayed a weak H‐1/H‐2 correlation while correlations from H‐2 to H‐6 were all intense, with H‐6 being a methyl at 1.24 ppm.

Integration of these data with those from the HSQC spectrum (Figure S3a), showed that C‐2 and C‐3 (78.9 and 82.2 ppm, respectively) were shifted at low field compared to the reference values because glycosylated,^[19]^ and that the anomeric carbon was α based on the diagnostic carbon chemical value of C‐5 (70.6 ppm).^[19]^ Finally, the NOESY spectrum (Figure S4c) correlated H‐1 of **B** with H‐2 of **A**, and H‐1 of **E** with H‐3 of **A**, this latter also confirmed by the corresponding correlation between H‐1 of **E** and C‐3 of **A** in the HMBC spectrum (labelled E_1_A_3_, Figure S3a).

The peak at about 5.00 ppm subtended about 5 anomeric signals (Figure S4b or 4c), however the one labeled **A′** (4.99 ppm) had a set of correlations in the NOESY spectrum very similar to that of **A**, which suggested that the two units were very similar. Indeed, the attribution of its proton and carbon chemical shifts through the combined used of TOCSY, COSY, and HSQC spectra, disclosed that **A′** was a 2,3‐linked Rha, attached to O‐3 of **B** and having **E′** linked to O‐3.

The COSY and TOCSY correlation pattern of **C** was similar to that of **A** and **A′**, indeed **C** was α rhamnose residue, and its carbon chemical shifts values indicated that it was α‐configured and linked at position 3, as proved by the low field C‐3 value (82.2 ppm), while the NOESY spectrum identified **D** as the unit linked at that position.

The residues **B** and **D** were identified, respectively as 3‐β‐GlcNAc and 3,4,6‐β‐GlcNAc. The *gluco* stereochemistry of **B** and **D** was deduced by the efficient magnetization transfer in the TOCSY spectrum, which displayed the correlations from H‐1 up to H‐5. The H‐2/C‐2 values of **B** and **D** (3.83/56.6 and 3.90/57.0 ppm, respectively) underlined the presence of an acetamido function, while each anomeric carbon was β configured, as proved by the H‐1/H‐3 and H‐1/H‐5 correlations in the NOESY spectrum (Figure S4c). Furthermore, the high chemical shift of the C‐3 signal of both residues (82.8 ppm for **B**, and 79.0 ppm for **D**, Figure S3a) revealed that **B** and **D** were glycosylated at the corresponding hydroxyl function, in agreement with the correlation between H‐3 of **B** and H‐1 of **A**, and H‐3 of **C** and H‐1 of **D**, visible in the NOESY spectrum (Figure S4c). **B** was not substituted at other positions, while C‐4 (75.8 ppm) and C‐6 (65.2 ppm, read by increasing the intensity of the HSQC spectrum, Figure S3c) of **D** denoted that this unit was further substituted even though the nature of the substituent could not be ascertained because of the lack of the appropriate correlations in the NOESY spectrum. However, the presence of the methyl of a pyruvic acid (^1^H/^13^C 1.47/26.2 ppm) suggested that these two positions were blocked with a pyruvate in the acetal form, with the *S* configuration based on the comparison of its chemical shifts (1.47/26.0 ppm) with those reported in literature;^[20]^ C‐2 and C‐1 values of the pyruvic group were read on the HMBC spectrum (Figure S3b and d).

Residue **E** was 2OMe‐β‐Vio4NAc linked to O‐3 of **A** as inferred from the key correlations in the NOESY (Figure S4c) and in the HMBC (Figure S3a) spectra. Almost coincident with H‐1 of **E** (4.59 ppm), there was a minor form of β‐Vio4NAc, labelled **E′** (H‐1 at 4.58 ppm), which differed from **E** by the lack of the methyl group at position 2, as deduced by the fact that its C‐2 value (75.0 ppm) was not shifted at low field as the other (84.3 ppm). This Vio4NAc unit was linked to O‐3 of **A′**, and its ratio versus the methylated form was calculated by comparing the H‐1/H‐2 densities in the TOCSY spectrum between **A** and **A′**, or between **E** and **E′**, which gave an averaged value of 4.7:1, that indicated that 80 % of Vio4NAc units were methylated in O‐2.

Taken together, the NMR study detected two different glycans in the fibrils. Polysaccharide 1 (poly_1) was composed by rhamnose (unit **C**) and N‐acetyl‐glucosamine (**D**) arranged in a linear repeating unit: 3)‐α‐L‐Rha‐(1→3)‐β‐D‐GlcNAc‐(1→, with the positions 4,6 of GlcNAc locked by a pyruvic acid linked as ketal (structure in Figure 2). Polysaccharide 2 (poly_2, structure in Figure 2) instead had a branched repeating unit, with rhamnose (unit **A** or **A′**) and N‐acetyl‐glucosamine (**B**) constructing the disaccharide repeat of the backbone: 2)‐α‐L‐Rha‐(1→3)‐β‐D‐GlcNAc‐(1→. The rhamnose unit was further glycosylated and it was labeled **A** when it carried a β‐D‐VioNAc unit methylated at O‐2 (unit **E**), or **A′** when the viosamine was not methylated (unit **E′**), with the ratio between the two forms of viosamine about 80 % in favor of **E**. Finally, the poly_1: poly_2 ratio was 1:3.1 (Table 1) as determined by comparing the intensity of the pyruvic acid (1.47 ppm), representative of poly_1, with that of the methyl signal at 1.24 ppm, related to poly_2, once corrected for the contribution of the other glycan.

**Table 1:** poly_1: poly_2 ratio in fractions purified by anion exchange chromatography. Polysaccharides ratios (poly_1/poly_2, here indicated as p1/p2) are evaluated by NMR integration of the appropriate signals; the yields (% mg mg^−1^) are calculated versus the amount of the starting material. Fractions eluted with NaCl concentration above 400 mM did not contain relevant amounts of carbohydrate material by NMR inspection and were not considered further.

		Untreated fibrils	Single treatment	Double treatment
Starting material	p1/p2	1.0: 3.1	1.0: 3.1	1.0: 3.1
	Mg	10.0	9.0	14.5
Sample loaded^[a]^	p1/p2	1.0: 3.1	1.0: 2.3	1.0: 1.4
	Mg	10.0	5.0	5.0
[NaCl]_mM_				
10	p1/p2	1.0: 4.8	1.0: 10.4	1.0: 10.4
	yield	2.7	1.4	1.9
100	p1/p2	1.0: 5.1	1.0: 5.1	1.0: 4.9
	yield	3.7	5.0	2.6
200	p1/p2	1.0: 2.3	1.0: 1.8	1.0: 1.7
	yield	2.3	2.0	4.2
400	p1/p2	1.0: 1.6	1.0: 0.82	1.0: 0.51
	yield	5.0	3.2	3.5
Total	yield	13.7	11.7	12.2

[a] the decrease of poly_1: poly_2 ratio from 3.1 in the intact fibrils to 1.4 in the double digested sample was explained by assuming that some of the poly_2 released by protease treatment was small enough to cross the dialysis membrane (3500 Da cut‐off) after the protease digestion.

### Purification and Molecular‐Weight Evaluation of the Two Glycans

The identification of two polysaccharides, one acidic (poly_1) and one with no ionizable groups (poly_2), made possible their separation by anion exchange chromatography.

Thus, the intact fibrils were separated by increasing the ionic strength of the eluent, and ^1^H NMR monitoring of the fractions found carbohydrate‐related material only in the eluates with 400 mM NaCl or less. Evaluation of the proportion between the two glycans, revealed that poly_2 was recovered in a rather enriched form in the fractions eluted with 10 and 100 mM NaCl (about five‐fold compared to poly_1), while the proportion of poly_1 increased in the others (200 and 400 mM NaCl), reaching a poly_1/poly_2 ratio of 1/1.6 at 400 mM NaCl (Table 1, Figure S5).

Based on the NMR evidence that the fibrils contained proteins and that these could interfere with the separation, this same purification procedure was repeated on the remaining of the fibrils post‐digestion with proteinase K. Consequently, the purity of each glycan increased, (Table 1, Figure S5), with poly_2 reaching a ten‐fold excess versus poly_1 in the fraction at the lowest ionic strength.

To further improve the separation between the two species, fibrils were treated twice with proteinase K and the same purification repeated. In this way, the purity of poly_2 presented the same (about ten‐fold) excess with respect to poly_1. On the contrary, the purity of poly_1, in the 400 mM fraction increased when compared to the previous two purifications, and its proportion almost doubled that of poly_2. Overall, these findings suggested that one single digestion was not sufficient to recover the two glycans in enriched form, probably because the peptide bonds were poorly accessible to the enzyme due to the presence of the polysaccharide(s).

Then, the total recovery on the intact fibrils indicated that the glycan‐containing material was only a fraction (13.7 %) of the loaded material. Interestingly, the global yield of material after a single or a double protease digestion was almost the same (about 12 %), and less if compared to that of the untreated fibrils, indicating that the treatment removed some portions of the protein(s) onto which the glycans were attached. This resizing of the proteic part by protease digestion enabled a better separation between the two glycans, thus suggesting that the two glycans were not interconnected anymore by the same polypeptide backbone.

**Figure 3:**
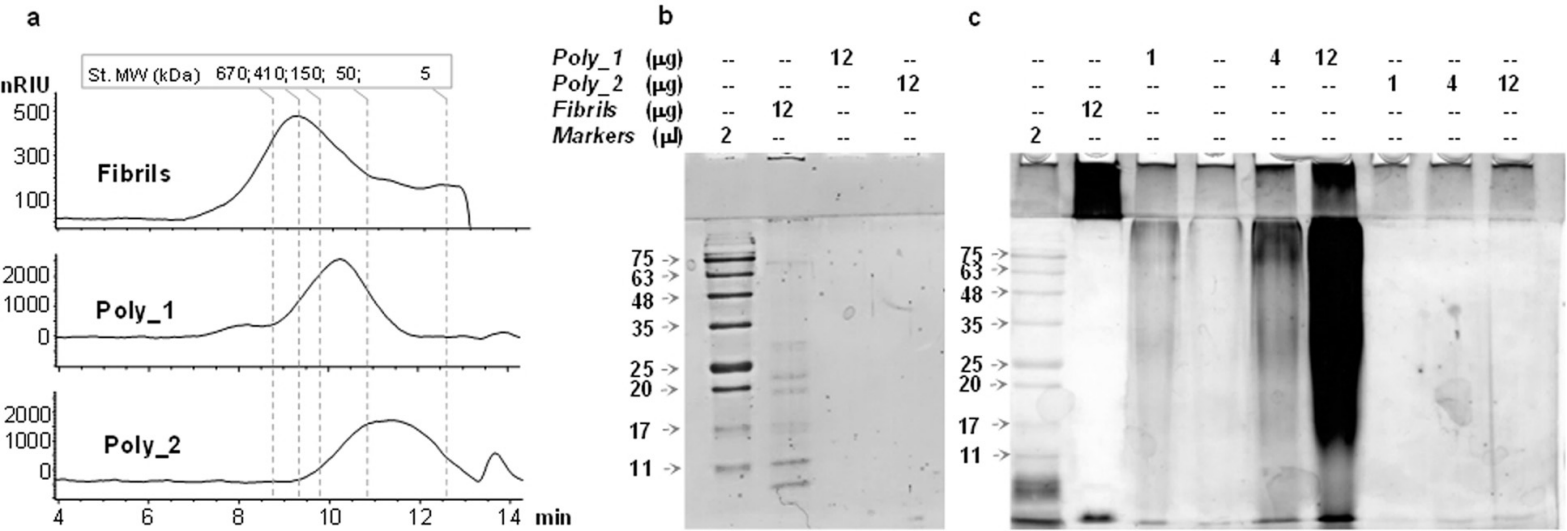
MW and SDS‐PAGE analysis of fibrils, poly_1 and poly_2. a) Size exclusion chromatographic profile detected by refractive index of the intact fibrils and of poly_1 and poly_2 isolated after double protease treatment, with the indication of the retention times of the dextran standards (St.) used to calibrate the column; b) and c) 12.5 % SDS‐PAGE profile of fibrils, poly_1 and poly_2 (from the double proteinase K treatment) stained for proteins (in b) or for acidic carbohydrates (in c).

The polysaccharides purified after a double protease digestion were then used to evaluate their molecular weight (MW) by size exclusion chromatography. As a result, poly_1 (from 400 mM NaCl fraction) and poly_2 (from 10 mM NaCl fraction) were 100 and 25 kDa, respectively, and each glycan presented a polydisperse distribution with the MW maximum centered to values lower than that of the untreated fibrils (400 kDa, Figure 3 a).

Finally, NMR analysis of the purified glycans confirmed the evaluations originally made on the intact fibrils (proton spectra in Figure 2, HSQC spectra in Figure S6). Interestingly, the HSQC spectrum of the two glycans reported the correlations for many of the minor signals previously poorly detected in the intact fibrils. These were monosaccharide units, none of them as N‐linked based on the ^13^C chemical shifts of the anomeric densities, likely related to previously reported oligosaccharides,^[21]^ although we cannot exclude that they, or some of them, are directly linked to the protein carrier through an *O*‐linkage. However, no other information could be deduced with confidence because of the complexity of the 2D spectra, and the overlap with the more intense signals of the glycans.

To rule out the possibility that the observed high MW of the fibrils could be due to unspecific aggregation, the different fractions were analyzed by SDS‐PAGE in denaturing conditions by applying a staining procedure selective for proteins (Figure 3 b) or for acidic polysaccharides (Figure 3 c). When the gel was stained to detect proteins, the intact fibrils (no proteinase K treatment) displayed only few bands in the separating gel (Figure 3 b). However, when the staining selective for acidic glycans was applied (Figure 3 c), the intact fibrils gave an intense response localized in the stacking gel, namely at very high molecular weights. This finding opposed to the MW of 100 kDa found for poly_1, and this discrepancy was solved by hypothesizing that poly_1 was covalently linked to a protein so that the MW of the whole ensemble was high enough to prevent its penetration into the separating gel, along its detection by conventional protein staining. In line with this model, the migration expected for poly_1 as a smear in the separating gel, was rescued upon digestion of the fibrils with proteinase K (Figure 3 c), thus supporting the idea that this glycan was covalently linked (in one or more copies) to at least one carrier protein, like in some eukaryotic proteoglycans or archaeal glycoproteins.^[1]^

Any attempt to visualize poly_2 by carbohydrate staining failed even at highest sample load (Figure 3 c). However, this negative result enabled us to rule out the anchoring of poly_2 to a lipid carrier, because in such case the glycan should have been detected as happens for lipopolysaccharides or lipoteichoic acids. Based on the conclusion reached for poly_1 and on the evidence that poly_2 purity increased only by repeating the protease digestion, it was assumed that poly_2 was also covalently linked to a carrier protein.

### Proteomic Analysis of Intact and Purified Fibrils

To get some clues about the set of proteins that could act as carriers for these two polysaccharides, we applied a nanoLC‐MS/MS proteomic approach by conventional shotgun analysis on three different samples: the full virus (positive control), the defibrillated virus (negative control), and the fibrils only.

This approach identified 181 different proteins, 160 being of viral origin (Source Data 1). This unexpected high number is probably due to i) the procedure to isolate the fibrils that induced a minor breakdown of the particles (about 10 %), and ii) the high sensitivity of mass spectrometry‐based proteomics that can detect minute amounts of proteins.

Hence, to uncover probable fibril proteins, we compared the spectral counts computed for each protein in the different samples (fibrils, defibrillated virus and full particle). An ACDtool analysis^[22]^ allowed to probabilistically sort out the proteins enriched in the fibril sample, which resulted in 29 proteins presenting the highest distances between fibrils and the defibrillated and full viruses (Source Data 1), namely representative of the fibrils composition.

**Figure 4:**
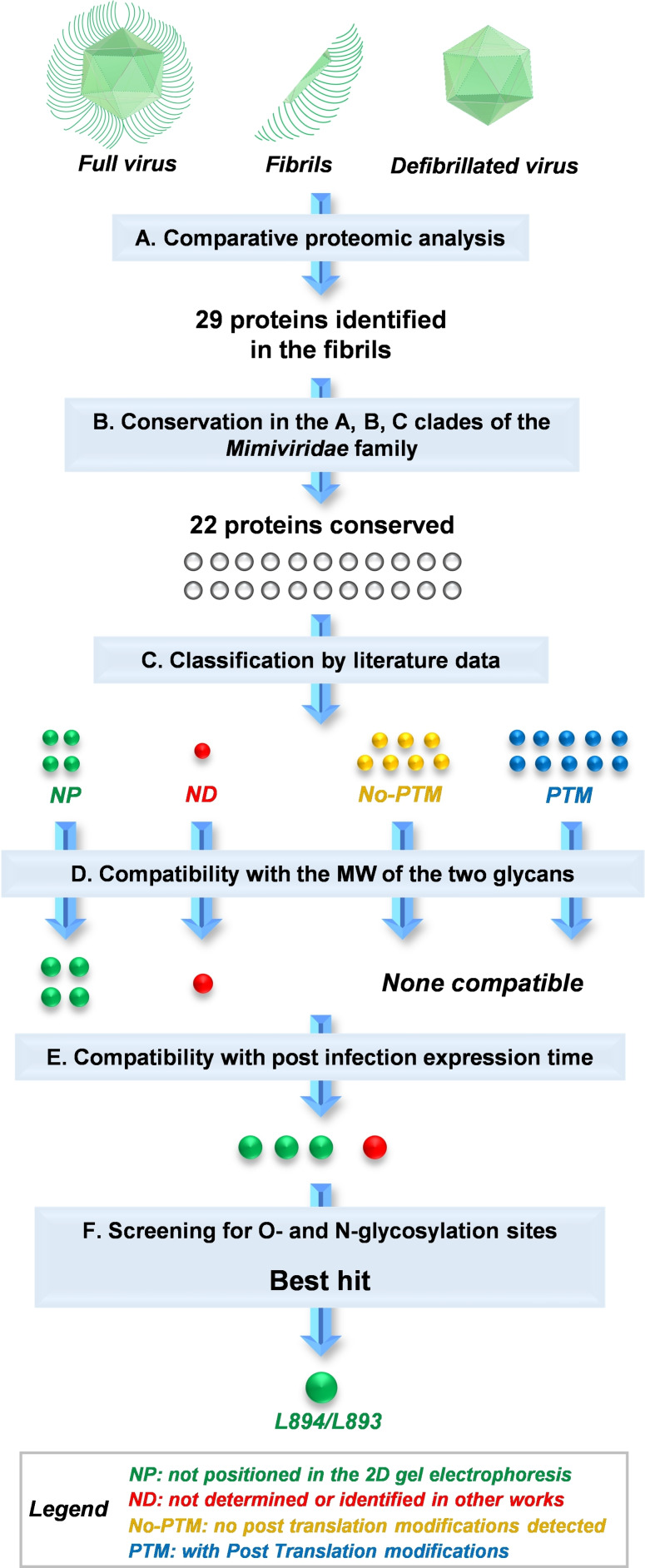
Strategy followed to define the potential carrier proteins of the two polysaccharides. A) Proteomic approach applied to the full virus, the fibrils and the defibrillated virus. B) Filter based on the protein conservation level through the A, B, and C clades of the *Mimiviridae* family. C) Sorting of the proteins as: modified by PTM (PTM), not modified (no‐PTM), not positioned (NP, no read‐out as with/without PTM from the experiment), and ND (data not available), based on literature data.^[10a]^ D) Compatibility of the proteins properties with the presence of any of the two glycans. E) Matching of the post‐infection expression time of the selected proteins with those associated to the glycosylation process. F) Evaluation of *N*‐ and *O*‐glycosylation sites with NetN/OGly Server. L894/L893 is a likely candidate, although the three in step E cannot be excluded since they might be glycosylated in sequons outside the prediction capability of the program.

These 29 proteins were thus considered as potential components of fibrils (Figure 4, step A).

Unfortunately, all the attempts made to determine the location of the glycosylation sites did not lead to conclusive results. First, we could not detect any glycopeptide fragment informative about the nature of the linkage and of the protein(s) involved. Second, any attempt to de‐glycosylate the fibrils (either targeting N‐ or O‐linked glycans) did not identify peptides carrying a specific mass shift due to deglycosylation, probably because the (enzymatic) tools used were not appropriate for these samples.

Then, to gain insight into which protein(s) could act as carrier, we resorted to a combination of bioinformatic tools with literature data.

First (Figure 4, step B), to restrict the pool of the potential carriers, we used tBlastn to define the conservation level of these 29 candidates across all the fully sequenced members of the first three clades of the *Mimiviridae* family (Table S3). This analysis included M4, the strain lacking fibrils (Figure 1 f) and many of the glyco‐related genes,^[17]^ and excluded the fourth clade (*Tupanviruses*) because genetically more distant from the others. This strategy neglected purposely the other viruses could differ for their glycosylation pattern. This approximation was driven by the knowledge that in all *Chloroviridae*, the capsid proteins have a high level of conservation, despite the structure attached glycan changes depending on the virus considered.^[6]^

This analysis restricted the pool to 22 proteins that were highly conserved (above 50 % of coverage) in the three clades. Then (Figure 4, step C), these 22 proteins were sorted by considering the information reported by Renesto et al.,^[10a]^ that applied a combination of 2D gel electrophoresis and MS experiments on the full particle of Mimivirus to define, among the identified proteins, those that were possibly bearing post‐translation modifications (PTMs).

Within our pool of 22 proteins, L236 was not detected by Renesto et al.,^[10a]^ and concerning the 21 left: 7 were devoid of PTMs, 10 had PTMs, while no information were deduced for the 4 remaining ones because the authors did not find them as positioned in the 2D gel electrophoresis (Table S2) used to assess if any PTM was occurring. Then (Figure 4, step D), the ten proteins with PTM were excluded from being the carriers of any of the two glycans, because their localization on the electrophoretic gel as a discrete number of spots was not compatible with the size and with the heterogeneous MW distribution found for these glycans in the current study. The PTMs observed by Renesto et al.,^[10a]^ seem compatible with small oligosaccharides, as those discovered from Hennet et al.,^[21]^ that we did not detect in our NMR study probably because they are minor components. Interestingly, for three of these proteins (R135, L829 and L725), it has been already demonstrated that they do not contribute to the hairy phenotype because their silencing leads to an abnormal shape of the fibrils but not to their disappearance.^[23]^

Accordingly (Figure 4, step D), this filter left the four proteins not positioned in the 2D gel electrophoresis (L894/L893, L488, L778 and R710),^[10a]^ and that undetected (L236) in the same work.

Then, we applied the post‐infection expression time as new selection criterion (Figure 4, step E). In this evaluation, we retained only the proteins whose expression was syntonic with the other proteins known to be involved in the sugar‐nucleotide metabolism (6–12 h post‐infection), considering this temporal coincidence as supportive of their role as carrier proteins. Thus, L788 was excluded, and the four remaining proteins (L894/L893, L488, R710 and L236), were screened for their predicted glycosylation pattern (Figure 4, step F).

The first to capture our attention was L894/L893 because absent in M4 (Table S2). L894/L893 is a big protein (702 aa) rich in serine, threonine and proline residues, and the NetOGly 4.0 Server,^[24]^ used for prediction of mucin O‐glycosylation sites, found ten sites, with three (^200^S, ^222^S and ^602^T) common in all the clades by multiple alignment (Figure S7). Of these three sites, the first two were detected by MS analysis of the tryptic digest, indicating that they were not modified by any PTM process. On the contrary, the last one was not found, supporting that L894/L893 could be decorated with one of the two polysaccharides.

Regarding L488, it is 559 aa long, still present in M4. This protein contains by prediction 50 O‐glycosylation sites of which 7 are preserved among the three clades (^197^S/T, ^374^S, ^506^T, ^511^T, ^518^T/S, ^523^T, ^540^T/S, Figure S8), all detected by MS analysis, except the last, ^540^T/S, probably because located in a tryptic peptide too small to be recorded or because glycosylated. Regarding the other two proteins, R710 and L236, they have no O‐glycosylation sites and only one asparagine residue in the classical sequon suitable for N‐glycosylation.

Based on the collective evaluation, our hypothesis is that L894/L893 is a likely carrier of one of the two polysaccharides. However, we cannot rule out completely the other three proteins or the glycosylation of L894/L893 in additional (and undetected) positions.

Indeed, all these proteins may possess glycosylation sites that have escaped the software prediction, since this software was optimized for mammal's glycosylation patterns.

Moreover, both *N*‐ and *O*‐glycosylation may occur in non‐canonical positions, therefore invisible to any software available, as demonstrated for the capsid proteins of *Chloroviridae*.^[6]^

Additional work will be needed to solve the challenge related to the glycosylation pattern and to precisely elucidate the fibrils structural network and composition.

### Discussion

In this study, the combined use of chemical, spectroscopic and spectrometric approaches has enabled us to shed light on the nature of the fibrils of Mimivirus.

We have indeed discovered that the fibrils are composed of a complex set of proteins, with one—L894/L893—likely glycosylated with one of the two polysaccharides, poly_1 and poly_2 (Figure 2) of 100 and 25 kDa, respectively. This protein was not annotated as glycosylated previously, and its function is still uncharacterized.

With regard to the two polysaccharides, they have no equivalent in archaea (query on Bacterial Carbohydrate Structure Database, in March 2021),^[25]^ and eukaryotes, including its host, *Acanthamoeba castellani*.^[26]^ We could find some similarities with bacterial glycans only for poly_1.^[25]^ This polysaccharide is somehow comparable to the O‐antigens of some strains of *Klebsiella pneumoniae*,^[27]^ *Serratia marcescens* ^[27]^ and *Agrobacterium tumefaciens*,^[28]^ all lacking the pyruvate substituent. On the contrary, poly_2 has no equivalent with any reported bacterial structure. Interestingly, these glycans are likely synthesized by a glycosylation machinery encoded mostly, if not entirely, by the virus itself.[^13^, ^14^, ^15^] Indeed, all the proteins necessary for the production of the monosaccharides used to build the two polysaccharides are encoded by the viral genome, along with several glycosyltransferases, whose specificity is yet to be established.^[15b]^

However, the structural novelty of these two glycans is not the most remarkable finding of this study. As anticipated in the introductory section, for all the viruses endowed with a glycan shield, the size of the glycan is limited to 6–12 monosaccharides units.

Mimivirus is different: contrary to all these viruses, its glycocalyx is made of polysaccharides and thus resembles bacteria (Figures 1 a and b), in syntony with the visionary name “Mimivirus” given in occasion of its discovery, which stands for “mimicking microbe virus”.

Then, there is the intriguing question on the role of this glycocalix and the advantage it provides to the virus. It was initially proposed that the size of Mimivirus and the glycosylation of its fibrils were meant to mimic the outer surface of bacteria to gain an entry in the amoeba host, as amoeba are normally feeding on bacteria.^[9a]^ Indeed, the adherence to the host cells of M4, devoid of fibrils, is significantly reduced compared to the wild type strain.^[29]^ Therefore, this coating of the capsid seems an advantage in an environment where various viral particles are competing for the same host cell. Besides, such glycocalyx could play an additional role in protecting the viral capsid from the environment in which the virus needs to survive,^[12]^ as suggested by the fact that none of the environmental isolates are devoid of fibrils.

Finally, the way these two polysaccharides are branched on protein(s) is reminiscent of archaeal S‐layer, and eukaryotic proteoglycans. This finding could sign an ancestral origin of the Mimivirus glycosylation pathway, predating the radiation of eukaryotes, as already suggested by the phylogenetic analyses of the various enzymes involved in monosaccharide synthesis.[^14^, ^15b^] Giant viruses now become a new source of carbohydrate active enzymes, raising the question on their contribution to the evolution of glycans pathways and polysaccharides synthesis.

## Conclusion

This study has addressed the nature of the fibrils of Mimivirus, demonstrating that this virus coats its surface with polysaccharides, and not with oligosaccharides of modest length as instead reported hitherto for all viruses.

This finding breaks the dogma according to which, such macromolecules present in all realms of life were absent from viruses, and it teach us that there is still a lot to learn by studying them. The glycosylation pattern of giant viruses is one of the facets still widely unexplored that must be considered to fully understand the physiology of these entities.

## Conflict of interest

The authors declare no conflict of interest.

## Supporting information

As a service to our authors and readers, this journal provides supporting information supplied by the authors. Such materials are peer reviewed and may be re‐organized for online delivery, but are not copy‐edited or typeset. Technical support issues arising from supporting information (other than missing files) should be addressed to the authors.

Supporting Information

Supporting Information

## References

[1] A. Varki , Glycobiology 2017, 27, 3–49. 27558841 10.1093/glycob/cww086 PMC5884436

[2] S. A. Jeffers , D. A. Sanders , A. Sanchez , J. Virol. 2002, 76, 12463–12472. 12438572 10.1128/JVI.76.24.12463-12472.2002 PMC136726

[3] M. Pabst , M. Chang , J. Stadlmann , F. Altmann , Biol. Chem. 2012, 393, 719. 22944675 10.1515/hsz-2012-0148

[4] J. Van Etten , I. Agarkova , D. Dunigan , M. Tonetti , C. De Castro , G. Duncan , Viruses 2017, 9, 88. 28441734 10.3390/v9040088 PMC5408694

[5] C. De Castro , A. Molinaro , F. Piacente , J. R. Gurnon , L. Sturiale , A. Palmigiano , R. Lanzetta , M. Parrilli , D. Garozzo , M. G. Tonetti , J. L. Van Etten , Proc. Natl. Acad. Sci. USA 2013, 110, 13956–13960. 23918378 10.1073/pnas.1313005110 PMC3752267

[6] C. De Castro , I. Speciale , G. Duncan , D. D. Dunigan , I. Agarkova , R. Lanzetta , L. Sturiale , A. Palmigiano , D. Garozzo , A. Molinaro , M. Tonetti , J. L. Van Etten , Angew. Chem. Int. Ed. 2016, 55, 654–658; 10.1002/anie.201509150 PMC4836869 26582281

[7] C. F. Quispe , A. Esmael , O. Sonderman , M. McQuinn , I. Agarkova , M. Battah , G. A. Duncan , D. D. Dunigan , T. P. L. Smith , C. De Castro , I. Speciale , F. Ma , J. L. Van Etten , Virology 2017, 500, 103–113. 27816636 10.1016/j.virol.2016.10.013 PMC5127778

[8] I. Speciale , I. Agarkova , G. A. Duncan , J. L. Van Etten , C. De Castro , Antonie van Leeuwenhoek 2017, 110, 1391–1399. 28331984 10.1007/s10482-017-0861-3

[9a] D. Raoult , Science 2004, 306, 1344–1350; 15486256 10.1126/science.1101485

[9b] B. L. Scola , Science 2003, 299, 2033–2033. 12663918 10.1126/science.1081867

[10a] P. Renesto , C. Abergel , P. Decloquement , D. Moinier , S. Azza , H. Ogata , P. Fourquet , J.-P. Gorvel , J.-M. Claverie , J. Virol. 2006, 80, 11678–11685; 16971431 10.1128/JVI.00940-06 PMC1642625

[10b] M. Legendre , S. Audic , O. Poirot , P. Hingamp , V. Seltzer , D. Byrne , A. Lartigue , M. Lescot , A. Bernadac , J. Poulain , C. Abergel , J. M. Claverie , Genome Res. 2010, 20, 664–674. 20360389 10.1101/gr.102582.109 PMC2860168

[11] W. Commons, Bacillus subtilis.jpg—Wikimedia Commons, the free media repository, available at: https://commons.wikimedia.org/w/index.php?title=File:Bacillus subtilis.jpg&oldid=494581146 **2020**.

[12] Y. G. Kuznetsov , C. Xiao , S. Sun , D. Raoult , M. Rossmann , A. McPherson , Virology 2010, 404, 127–137. 20552732 10.1016/j.virol.2010.05.007

[13] M. Parakkottil Chothi , G. A. Duncan , A. Armirotti , C. Abergel , J. R. Gurnon , J. L. Van Etten , C. Bernardi , G. Damonte , M. Tonetti , J. Virol. 2010, 84, 8829–8838. 20538863 10.1128/JVI.00770-10 PMC2918987

[14] F. Piacente , C. Bernardi , M. Marin , G. Blanc , C. Abergel , M. G. Tonetti , Glycobiology 2014, 24, 51–61. 24107487 10.1093/glycob/cwt089

[15a] F. Piacente , C. De Castro , S. Jeudy , M. Gaglianone , M. E. Laugieri , A. Notaro , A. Salis , G. Damonte , C. Abergel , M. G. Tonetti , J. Biol. Chem. 2017, 292, 7385–7394; 28314774 10.1074/jbc.M117.783217 PMC5418040

[15b] F. Piacente , M. Marin , A. Molinaro , C. De Castro , V. Seltzer , A. Salis , G. Damonte , C. Bernardi , J.-M. Claverie , C. Abergel , M. Tonetti , J. Biol. Chem. 2012, 287, 3009–3018. 22157758 10.1074/jbc.M111.314559 PMC3270958

[16] C. Xiao , Y. G. Kuznetsov , S. Sun , S. L. Hafenstein , V. A. Kostyuchenko , P. R. Chipman , M. Suzan-Monti , D. Raoult , A. McPherson , M. G. Rossmann , PLoS Biol. 2009, 7, e1000092. 19402750 10.1371/journal.pbio.1000092 PMC2671561

[17] M. Boyer , S. Azza , L. Barrassi , T. Klose , A. Campocasso , I. Pagnier , G. Fournous , A. Borg , C. Robert , X. Zhang , C. Desnues , B. Henrissat , M. G. Rossmann , B. La Scola , D. Raoult , Proc. Natl. Acad. Sci. USA 2011, 108, 10296–10301. 21646533 10.1073/pnas.1101118108 PMC3121840

[18] T. Klose , D. A. Herbst , H. Zhu , J. P. Max , H. I. Kenttämaa , M. G. Rossmann , Structure 2015, 23, 1058–1065. 25982526 10.1016/j.str.2015.03.023 PMC4456301

[19] K. Bock , C. Pedersen , Adv. Carbohydr. Chem. Biochem. 1983, 41, 27–66.

[20] P. J. Garegg , P.-E. Jansson , B. Lindberg , F. Lindh , J. Lönngren , I. Kvarnström , W. Nimmich , Carbohydr. Res. 1980, 78, 127–132.

[21] A. J. Hülsmeier , T. Hennet , Glycobiology 2014, 24, 703–714. 24794008 10.1093/glycob/cwu034

[22] J.-M. Claverie , T. N. Ta , Bioinformatics 2019, 35, 170–171. 30020402 10.1093/bioinformatics/bty640

[23] H. Sobhy , B. L. Scola , I. Pagnier , D. Raoult , P. Colson , Front. Microbiol. 2015, 6, 00345. 10.3389/fmicb.2015.00345 PMC4412084 25972846

[24] C. Steentoft , S. Y. Vakhrushev , H. J. Joshi , Y. Kong , M. B. Vester-Christensen , K. T.-B. G. Schjoldager , K. Lavrsen , S. Dabelsteen , N. B. Pedersen , L. Marcos-Silva , R. Gupta , E. P. Bennett , U. Mandel , S. Brunak , H. H. Wandall , S. B. Levery , H. Clausen , EMBO J. 2013, 32, 1478–1488. 23584533 10.1038/emboj.2013.79 PMC3655468

[25] P. V. Toukach , K. S. Egorova , Nucleic Acids Res. 2016, 44, D1229–D1236. 26286194 10.1093/nar/gkv840 PMC4702937

[26] B. Schiller , G. Makrypidi , E. Razzazi-Fazeli , K. Paschinger , J. Walochnik , I. B. H. Wilson , J. Biol. Chem. 2012, 287, 43191–43204. 23139421 10.1074/jbc.M112.418095 PMC3527907

[27] I. A. Knirel’ , N. K. Kochetkov , Biokhimiya 1994, 59, 1784–1851. 7533007

[28] C. De Castro , A. Carannante , R. Lanzetta , R. Nunziata , V. Piscopo , M. Parrilli , Carbohydr. Res. 2004, 339, 2451–2455. 15388361 10.1016/j.carres.2004.07.017

[29] R. A. L. Rodrigues , L. K. dos Santos Silva , F. P. Dornas , D. B. de Oliveira , T. F. F. Magalhães , D. A. Santos , A. O. Costa , L. de Macêdo Farias , P. P. Magalhães , C. A. Bonjardim , E. G. Kroon , B. La Scola , J. R. Cortines , J. S. Abrahão , J. Virol. 2015, 89, 11812–11819. 26378162 10.1128/JVI.01976-15 PMC4645322

