# Supplementary material for: Expanding the Occurrence of Polysaccharides to the Viral World: The Case of Mimivirus
Source: Angew Chem Int Ed Engl. 2021 Aug 3;60(36):19897–904. doi: 10.1002/anie.202106671 (PMC8456856; doi:10.1002/anie.202106671)
Supplement: Supplementary file 1 — Supporting Information [file ANIE-60-19897-s002.pdf]

Angewandte Chemie
Eine Zeitschrift der Gesellschaft Deutscher Chemiker

Supporting Information

# Expanding the Occurrence of Polysaccharides to the Viral World: The Case of Mimivirus

*Anna Notaro, Yohann Couté, Lucid Belmudes, Maria Elena Laugeri, Annalisa Salis, Gianluca Damonte, Antonio Molinaro, Michela G. Tonetti,* Chantal Abergel,* and Cristina De Castro**

anie_202106671_sm_miscellaneous_information.pdf
anie_202106671_sm_Source_Data_1.xlsx

## Author Contributions

A.N. Data curation: Lead; Formal analysis: Lead; Funding acquisition: Lead; Writing—original draft: Lead
Y.C. Data curation: Lead; Funding acquisition: Equal; Investigation: Equal; Supervision: Equal; Writing—review & editing: Equal
L.B. Investigation: Equal
M.L. Investigation: Supporting
A.S. Investigation: Supporting
G.D. Investigation: Supporting
A.M. Conceptualization: Equal; Supervision: Equal; Writing—review & editing: Equal
M.T. Supervision: Equal; Writing—review & editing: Equal
C.A. Conceptualization: Lead; Funding acquisition: Lead; Investigation: Lead; Supervision: Equal; Writing—review & editing: Lead
C.D. Conceptualization: Lead; Funding acquisition: Lead; Investigation: Lead; Supervision: Lead; Writing—review & editing: Lead.

# SUPPORTING INFORMATION

## Table of Contents Page


| | | |
|---|---|---|
| 1. | Experimental Procedures | 2 |
| 1.1 | Mimivirus fibrils glycans extraction | 2 |
| 1.2 | TEM analysis of the viral particle and fibrils obtained from DTT treatment**.** | 2 |
| 1.3 | Sugar composition of Mimivirus fibrils and of the M4 strain | 2 |
| 1.4 | NMR data acquisition of fibrils | 2 |
| 1.5 | Separation of Mimivirus polysaccharides by anion exchange chromatography | 2 |
| 1.6 | Molecular weight determination | 3 |
| 1.7 | SDS-PAGE analysis | 3 |
| 1.8 | Mass Spectrometry-based proteomic analysis of Mimivirus fibrils, full virions and defibrillated virions | 3 |
| 1.9 | Conservation of fibrils proteins through the three clades of the *Mimiviridae* family | 3 |
| 1.10 | Determination of the putative glycosylation sites for L894/L893 and L488, and their conservation in other members | 3 |
| 2. | Supporting Figures and Tables | 4 |
| Figure S1. | Monosaccharide analysis via GC-MS | 4 |
| Figure S2. | GC-MS analysis of partially methylated alditol acetates of the fibrils. | 5 |
| Figure S3. | $^{1}$H and $^{13}$C heteronuclear spectra of Mimivirus fibrils. | 6 |
| Figure S4. | $^{1}$H-$^{1}$H Homonuclear spectra of Mimivirus fibrils. | 7 |
| Figure S5. | Evaluation of poly_1 *vs* poly_2 ratio. | 8 |
| Figure S6. | NMR data of purified poly_1 and poly_2. | 9 |
| Figure S7. | Structure-based alignment of L894/L893 (YP_003987467). | 10 |
| Figure S8. | Structure-based alignment of Structure-based alignment of L488 (YP_003986999) | 11 |
| Table S1. | NMR chemical shifts of poly_1 and poly_2. | 12 |
| Table S2. | Conservation level of the 29 proteins most enriched in the fibrils in the Acanthamoeba infecting *Mimiviridae* family. | 13 |
| Table S3. | Genome accession numbers used to derive the data in Supporting Table 2. | 14 |
| 3. | References | 15 |
| 4. | Authors contribution | 15 |
| 4. | Supplementary file. | |

Data 1. (Excel file) Mass spectrometry-based proteomic analyses of full virus, defibrillated virus and fibrils.

SUPPORTING INFORMATION

# 1. Experimental Procedures

### 1.1 Mimivirus fibrils glycans extraction.

*Acanthamoeba polyphaga mimivirus*, here named Mimivirus, and its M4 isolate were purified from infected *Acanthamoeba castellanii* culture as previously described.[1] Fibrils isolation was performed on Mimivirus by developing the conditions reported by Xiao et al.[2] with minor variations. Briefly, about 1.0 × $10^{11}$ viral particles were treated with 50 mM DTT (20 ml), 100°C for 2 h under stirring (100 rpm). The supernatant containing the fibrils was separated from the viral particles by centrifugation (10 min., 4650 g, room temperature), and an aliquot of both samples was used for TEM analysis. Then, the solid was washed twice with water (1 ml), the supernatants were pooled, dialyzed against water (membrane cut-off 3500 Da) to remove the DTT and frozen dried (9.3 mg).

### 1.2 TEM analysis of the viral particle and fibrils obtained after DTT treatment.

The untreated Mimivirus, the fibrils and the defibrillated virus obtained after DTT treatment, were compared by TEM to the M4 isolate. All the samples were fixed in glutaraldehyde 2.5% in water for 1h at room temperature. After fixing, the samples were centrifuged at 5000 g for 10 min and the pellets were washed twice with water.

The structure of the fibrils was visualized by negative staining using methyl cellulose (M6385 Sigma) and uranyl acetate 2% in water. In detail, 2 µl of each sample was adsorbed for 3 min on a Formarv® (CAS#63450-15-7) coated grid. Then the grid was treated with 2% uranyl acetate in water for 1 min. The excess of uranyl acetate was removed by quickly washing the grid with a drop of a methyl cellulose solution (200 µl 2% uranyl acetate plus 1800 µl of 2% methyl cellulose in water). Next, the grid was stained with this same solution for 1 min and 30 seconds, and the excess of methyl cellulose was removed by drying the grid on a paper filter. All the samples were observed on TEM TECNAI G2°200 KV.

### 1.3 Sugar composition of Mimivirus fibrils and of M4 strain.

Monosaccharide composition analysis as acetylated methyl glycoside was performed on the fibrils (0.5 mg) of Mimivirus or directly on the intact M4 viral particle, according to the protocol reported from De Castro et al.[3]

The identification of Rha, GlcNAc and other minor components (Fig. S1a) made use of the available commercial standards, after proper derivatization. Regarding VioNAc, and 2OMeVioNAc, the identification was inferred by applying the fragmentation rules described for these derivatives.[4] In particular, for the 2-*O*-methylated derivatives of VioNAc, the EI-MS spectrum (Figure S1b) contained a fragment at m/z 244 consistent with the oxonium ion of a 6-deoxy-aminosugar with two acetyls and one methyl group as substituents. The fragment at m/z 88 indicated that the methoxyl group was located next to the anomeric methoxyl group, in agreement with our previous NMR data.[5] These composition results are in agreement with the functional pathway for the UDP-L-Rha,[6] UDP-D-GlcNAc[7] and UDP-D-Vio4NAc.[5, 8]

The absolute configuration of the major sugar constituents was assumed to be L for Rha,[6] and D for GlcN[9] and Vio4NAc[5, 8] based on the biosynthetic data available for Mimivirus.

Linkage analysis of Mimivirus fibrils as partially methylated and acetylated alditol, was performed as reported.[3] GC-MS analyses were performed with an Agilent instrument (GC instrument Agilent 6850 coupled to MS Agilent 5973), equipped with a SPB-5 capillary column (Supelco, 30 m × 0.25 i.d., flow rate, 0.8 mL $min^{-1}$) and He as the carrier gas. Electron impact mass spectra were recorded with an ionization energy of 70 eV and an ionizing current of 0.2 mA. The temperature program used to analyze all the derivatives was: 150° C for 5 min, 150 → 280° C at 3° C/min, 300° C for 5 min.

### 1.4 NMR data acquisition of fibrils

1D and 2D NMR spectra of the fibrils prior any purification were measured on a Bruker 600 DRX equipped with a CryoProbe™ at 329 K to improve the quality of the spectra. Acetone was used as internal standard ($^{1}$H 2.225 ppm, $^{13}$C 31.45 ppm) and 2D spectra (COSY, TOCSY, NOESY, HSQC and HMBC) were acquired by using Bruker software (TopSpin 2.0). Homonuclear $^{1}$H-$^{1}$H 2D experiments were recorded using 512 FIDs of 2048 complex with 24 scans per FID, mixing times of 100 and 200 ms were used for TOCSY and NOESY spectra, respectively. $^{1}$H-$^{13}$C HSQC and HMBC spectra were acquired with 512 FIDs of 2048 complex point, accumulating 50 and 60 scans, respectively. As for the samples from ion exchange chromatography, all spectra were measured at 310 K, reduction of the solvent signal intensity was achieved by presaturation or by measuring a mono-dimensional DOSY spectrum. In this last case, spectra were recorded by setting $\delta$ and $\Delta$ to 2.4 µs and 100 ms, respectively, and the variable gradient to 45% of its maximum power. Spectra were processed and analysed using a Bruker TopSpin 3 program. 2D NMR spectra on pure poly_1 and poly_2 obtained after extensive proteinase K digestion and ion exchange purification, were measured at 310 K, as indicated for the untreated fibrils.

### 1.5 Separation of Mimivirus polysaccharides by anion exchange chromatography.

Fibrils either intact or after protease treatment(s) (5-10 mg) were purified by anion exchange chromatography using Q-Sepharose fast flow as adsorbent (0.5 ml of gel for 5-10 mg of fibrils) and fractions were collected by increasing stepwise NaCl concentration (10, 100, 200, 400, 700 and 1000 mM). Each solution was applied for 5 column volumes and eluates with the same ionic strength were collected together and desalted on a Biogel P10 column (20 ml, 12 ml/h) by using mq $H_2O$ as eluent and monitoring the eluate with an on-line refractive index detector (K-2310 Knauer). The polysaccharide material was eluted in the void volume of the column. $^{1}$H NMR was used to evaluate the ratio between the two polysaccharides which occurred by integration of the signals of interest: the methyl of the pyruvic acid at 1.47 ppm was selected as reporter group of poly_1, and the signal 1.24 ppm which included the methyl groups of rhamnose **C** of poly_1, rhamnose units **A** and **A'**, and 2OMeVio4NAc units **E** and **E'**, present in poly_2 (for labels

description refer to Supplementary Table 1). The following formula has been used to determine the proportion between the two polysaccharides:

$$poly_1 = \frac{A_{1.47ppm}}{A_{1.47ppm}};\ poly_2 = \frac{\frac{[A_{1.24ppm} - A_{1.47ppm}]}{2}}{A_{1.47ppm}}$$

Each protease digestion was performed treating the material with proteinase K (Sigma P6556-100 MG, CAS 39450-01-6), using 0.5 mg of the protease for 10 mg of sample in a digestion buffer (100 mM Tris, 50 mM NaCl, 10 mM $MgCl_2$, pH 7.5) at 55°C O.N. Then the samples were dialyzed against water (cut-off 3500 Da) and dried. Yields and poly_2/poly_1 ratio for the different purifications are reported in Table 1.

**1.6 Molecular weight determination**

The molecular weight was determined by size exclusion chromatography, by using a TSK gel G5000 PWXL column (30 cm x 7.8 mm ID), eluted with 50 mM ammonium bicarbonate, at 0.8 ml/min at r.t., the eluate was monitored with a refractive index detector on a HPLC system Agilent 1100. The column was calibrated with dextrans standards of known molecular weight (5, 50, 150, 410, and 610 kDa), the logarithm of the molecular weight was fitted versus the elution volume with a linear regression and the molecular weight of the fibrils, poly_1 and poly_2 was evaluated by interpolation of the equation.

**1.7 SDS-PAGE analysis**

The 12.0% SDS PAGE (Figures 4b and 4c) used to detect carbohydrate material by alcian blue and silver staining[3] was performed on the fibrils (12 μg), and on poly_1 (1, 4, 12 μg) and poly_2 (1, 4, 12 μg) with the highest purity. The BlueEye Prestained Protein marker (2 μl, Bioscience) was loaded as molecular weight reference. A similar gel was stained with Comassie brilliant blue to evidence the protein content, here poly_1 and poly_2 were used at the maximum concentration (12 μg each).

**1.8 Mass Spectrometry-based proteomic analysis of Mimivirus fibrils, full virions and defibrillated virions**

Extracted proteins were stacked in the top of a SDS-PAGE gel (4-12% NuPAGE, Life Technologies), stained with Coomassie blue R-250 (Bio-Rad) before in-gel digestion using modified trypsin (Promega, sequencing grade) as previously described.[10] Resulting peptides were analyzed by online nanoliquid chromatography coupled to tandem MS (UltiMate 3000 RSLCnano and Q-Exactive Plus, Thermo Scientific). Peptides were sampled on a 300 µm x 5 mm PepMap C18 precolumn and separated on a 75 µm x 250 mm C18 column (Reprosil-Pur 120 C18-AQ, 1.9 µm, Dr. Maisch) using a 120-min gradient. MS and MS/MS data were acquired using Xcalibur (Thermo Scientific). Peptides and proteins were identified using Mascot (version 2.6.0) through concomitant searches against Mimivirus database (homemade), classical contaminant database (homemade) and the corresponding reversed databases. The Proline software[11] was used to filter the results: conservation of rank 1 peptides, peptide score ≥ 25, peptide length ≥ 7, peptide-spectrum-match identification false discovery rate < 1% as calculated on scores by employing the reverse database strategy, and minimum of 1 specific peptide per identified protein group. Proline was then used to perform a compilation, grouping and spectral counting-based comparison of the protein groups identified in the different samples. Proteins from the contaminant database were discarded from the final list of identified proteins. Extracted spectral counts for each protein in each sample were tested using ACD tool (URL: www.igs.cnrs-mrs.fr/acdtool/).[12] Only proteins with a p-value inferior to 0.001 were considered as enriched in one of the compared samples.

**1.9 Conservation of fibrils proteins through three clades of the *Mimiviridae* family.**

Tblastn (https://blast.ncbi.nlm.nih.gov/Blast.cgi) was used to assess the presence, and the level of conservation (Table S2), of the restricted pool of 29 proteins *versus* all fully sequenced genomes of the members of the first three clades of the *Mimiviridae* family (Table S3). For each protein, the % of query coverage and identity is reported.

**1.10 Determination of putative glycosylation sites for L894/L893, and L488 and their conservation in other members.**

NetOGly 4.0 Server or NetNGlyc 1.0 available at DDTU Health Tech site (http://services.healthtech.dtu.dk) were used to identify the potential O- and N-glycosylation sites of: L894/L893, L488, and R710, and L236.

Contrary to the others, L894/L893 and L488 have several glycosylation sites and for this reason, only these two proteins were further compared with their orthologs in the three clades by using multiple alignment based on structural information on the Expresso Server (http://tcoffee.crg.cat/apps/tcoffee/do:expresso)[13] to define which glycosylation sites were conserved. The obtained multiple alignment was submitted to ESPript server (http://espript.ibcp.fr/ESPript/ESPript/)[14] to visualize sequence similarities (Figures S7 and S8, for L894/L893 and L488, respectively).

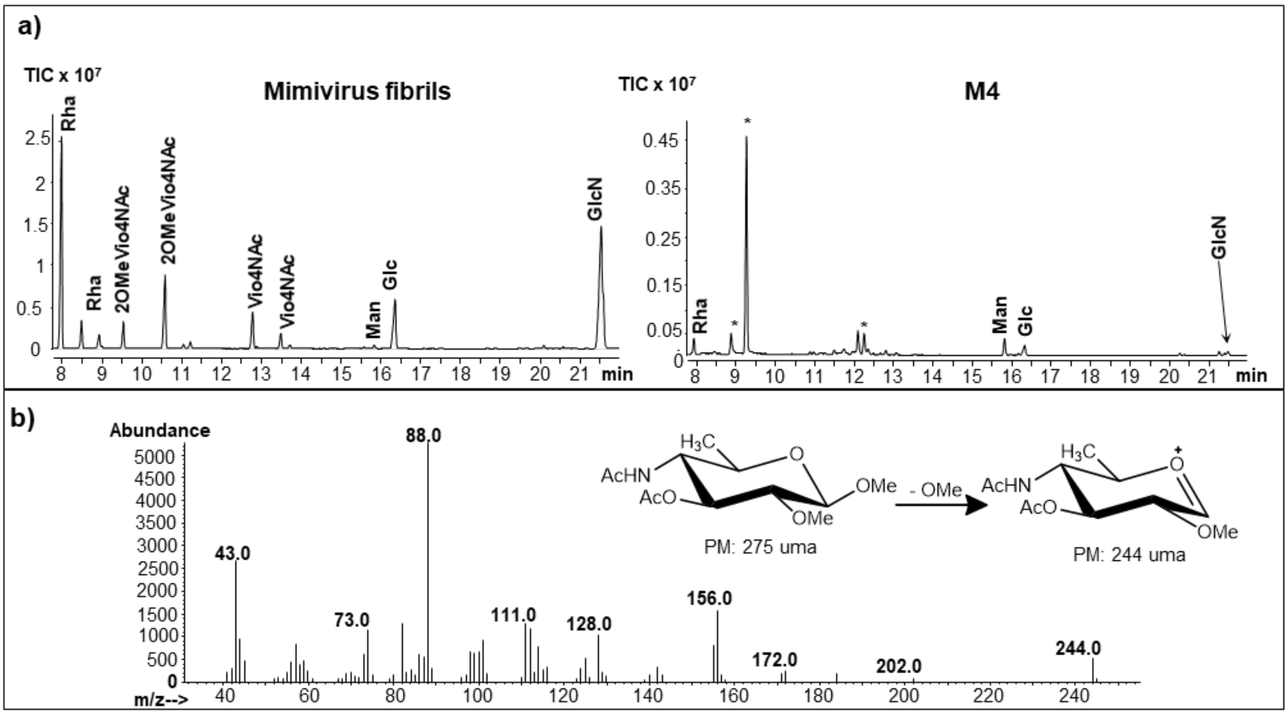


**Figure S1.** Monosaccharide analysis via GC-MS. a) monosaccharide profiles of the fibrils of Mimivirus (left) and of the whole M4 particle (right) the Mimivirus isolate devoid of fibrils. b) EI-MS spectrum of the 2OMeVio4NAc. The fragment at m/z 244 is consistent with the oxonium ion of a 6-deoxy-aminosugar substituted with a methyl group, and the fragment at m/z 88 reports two vicinal methoxyl groups, indicating that the anomeric methoxyl group is flanked by another at position two. * impurity.

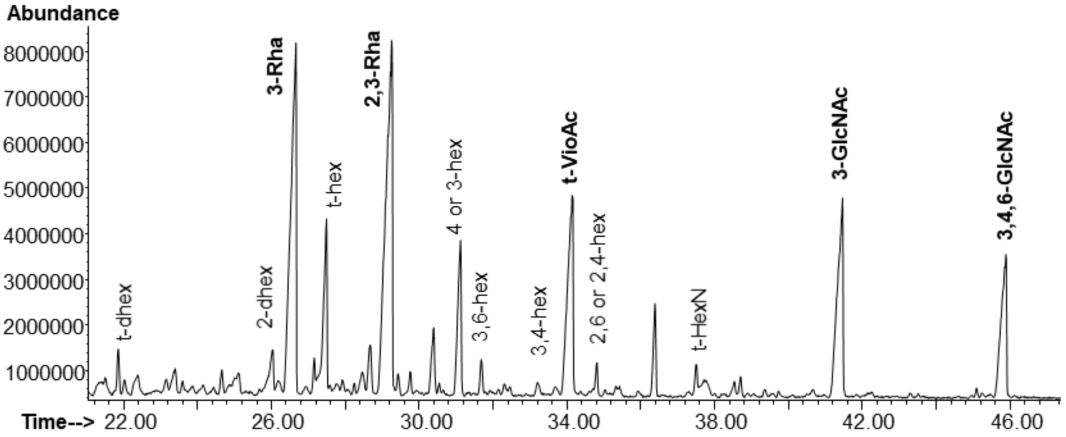


**Figure S2.** GC-MS analysis of partially methylated alditol acetates of the fibrils. The derivatives of the main sugars are written in bold: 3-Rha (Rha linked at position 3), 2,3-Rha, t-Vio4NAc, 3-GlcNAc and 3,4,6-GlcNAc. The stereochemistry of the other minor constituents was not determined.

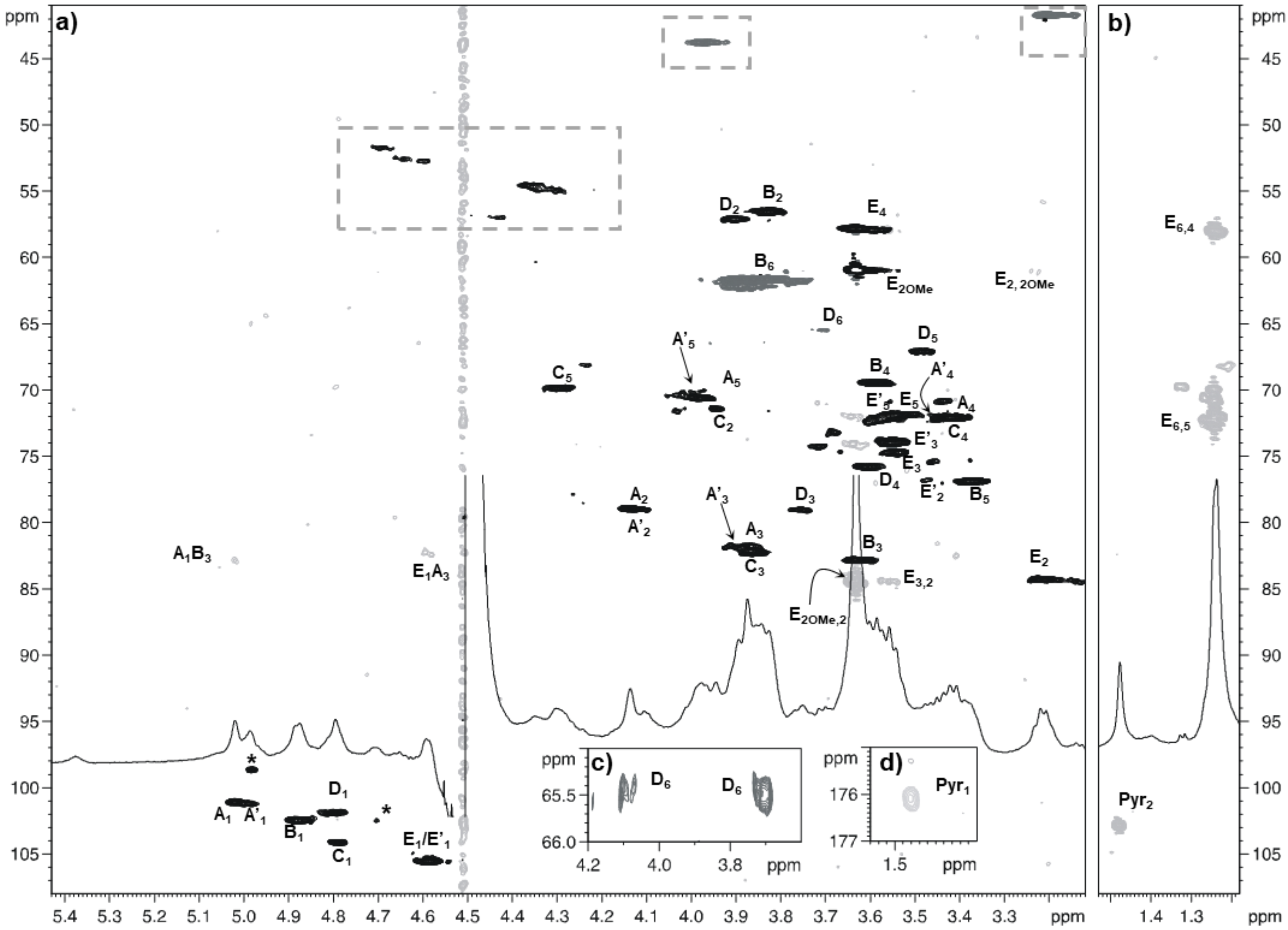

**Figure S3.** $^{1}H$ and $^{13}C$ heteronuclear spectra of *Mimivirus* fibrils. Overlay of HSQC (black/dark grey) and HMBC (light grey) spectra detailing: a) the anomeric and the carbinolic regions; b) the high field region containing the methyl signals, c) the D6 densities drawn by raising the contour levels, and d) the long range correlation connecting the methyl group of the pyruvate to the carboxylic carbon. Letters used for the annotation of the densities follow the system of Table S1. Signals in the dotted box are related to proteins and signals denoted with "*" refer to anomeric protons of minor monosaccharide motifs.

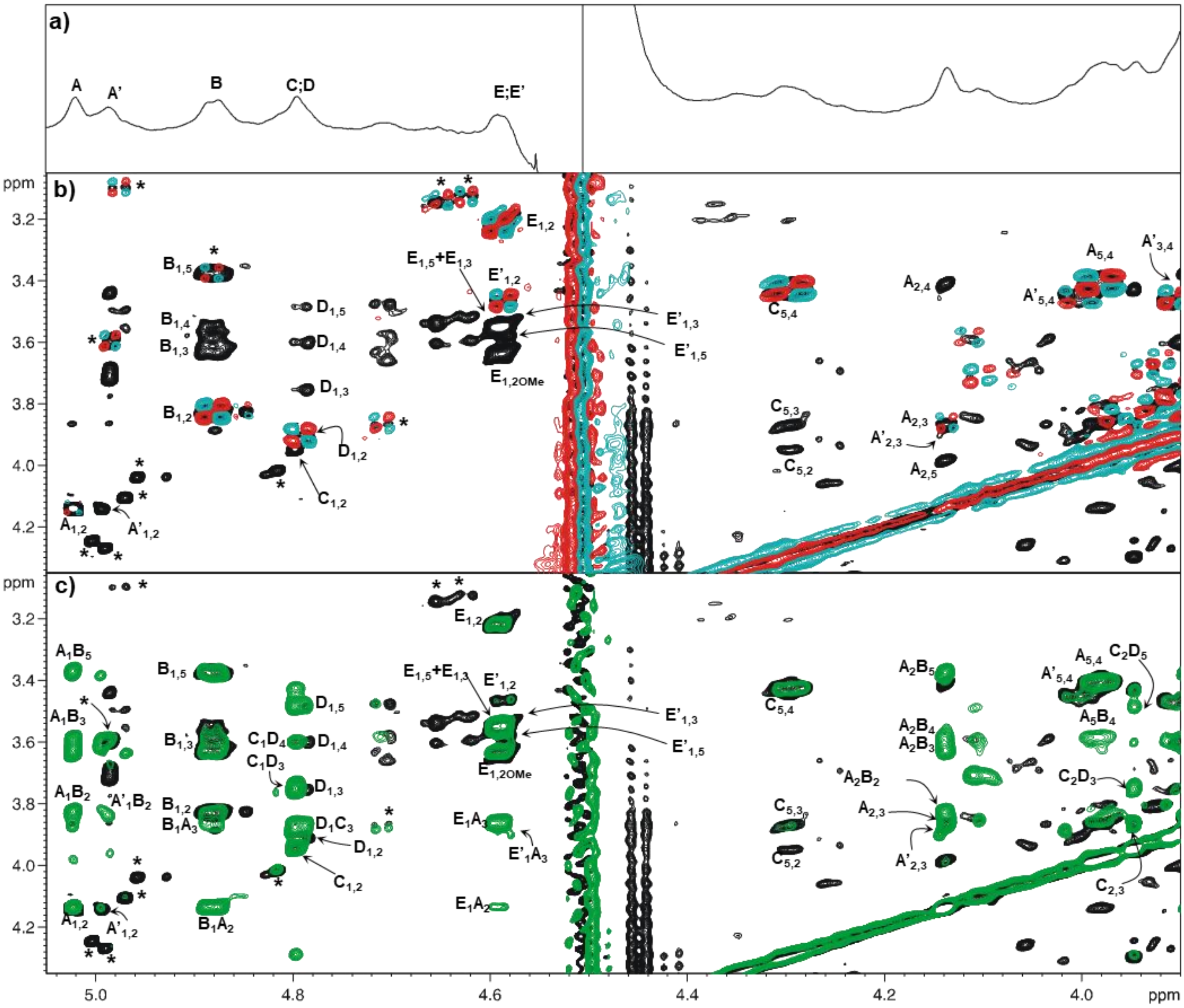

**Figure S4**. $^1$H-$^1$H Homonuclear spectra of Mimivirus fibrils. a) proton spectrum; b) overlay of TOCSY (black) and COSY (red and cyan) spectra; c) overlay of TOCSY (black) and NOESY (green) spectra. Letters used for the annotation of the densities follow the system of Table S1. *minor anomeric signals whose identity could not be established.

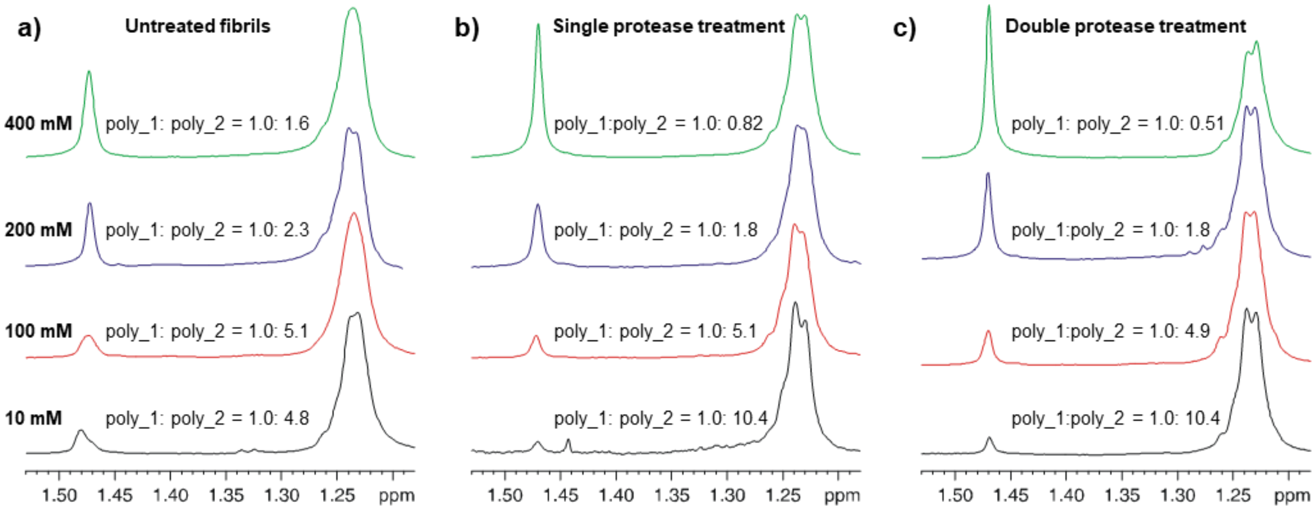


**Figure S5.** Evaluation of poly_1 vs poly_2 ratio. The methyl at $^{1}$H 1.47 ppm was used as reporter group of poly_1 and the group of signals at 1.24 ppm was related to poly_2 after subtraction of the contribution of poly_1. Integrations of the proton spectra were run for the all the fractions obtained by ion exchange chromatography and are reported next to each profile or in Table 1. a) untreated fibrils; b) fibrils digested with protease K; c) fibers digested twice with proteinase K.

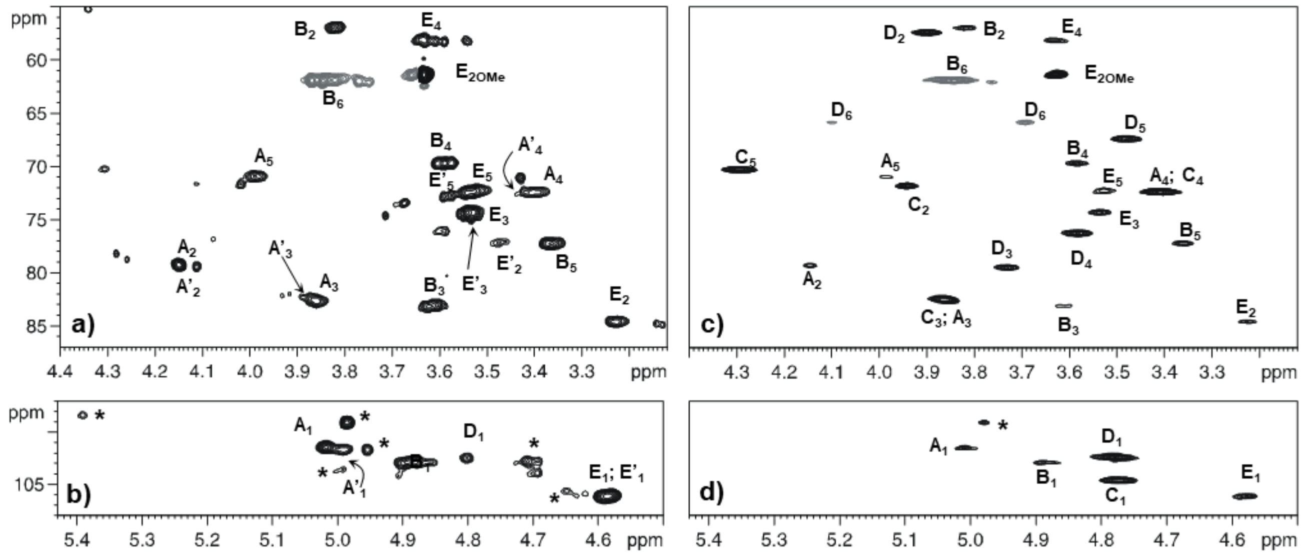

**Figure S6.** NMR data of purified poly_1 and poly_2. HSQC spectra of poly_2 and of poly_1 purified by ion exchange chromatography after the double proteinase K treatment; a) carbinolic region and b) anomeric region of purified poly_2; c) and d) the same for poly_1. Labels used to annotate the densities follow the system of Table S1, signals denoted with “*” refer to anomeric protons of minor monosaccharide motifs.

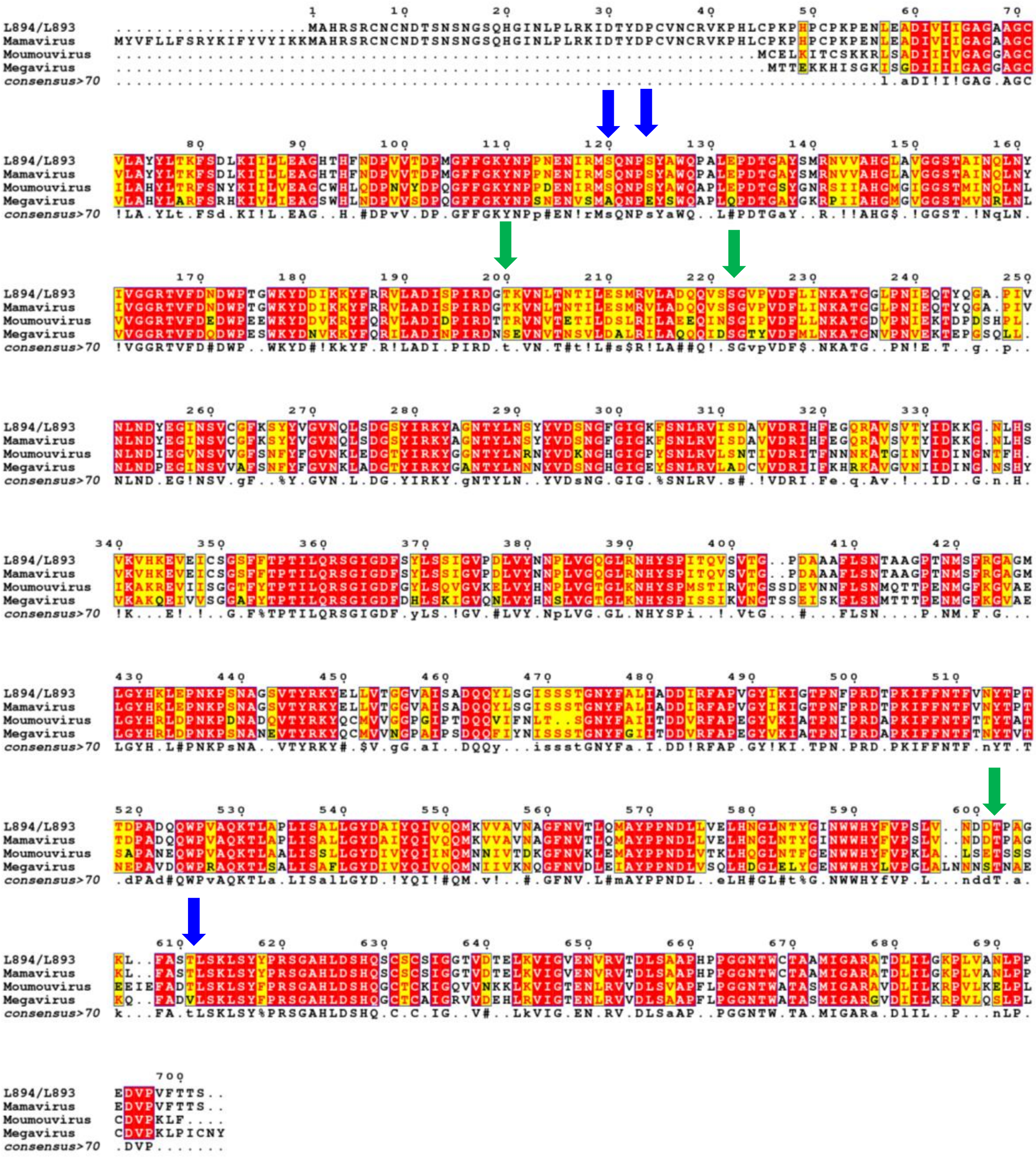


**Figure S7.** Structure-based alignment of L894/L893 (YP_003987467) with its orthologs in the A clade (Mamavirus- AEQ61113), B clade (Moumouvirus-YP_007354752) and C clade (Megavirus chilensis-YP_004894929). The green arrow points to conserved residues potentially involved in O-glycosylation. The blue arrow indicates potentially O-glycosylation sites preserved only in the A and B clades.

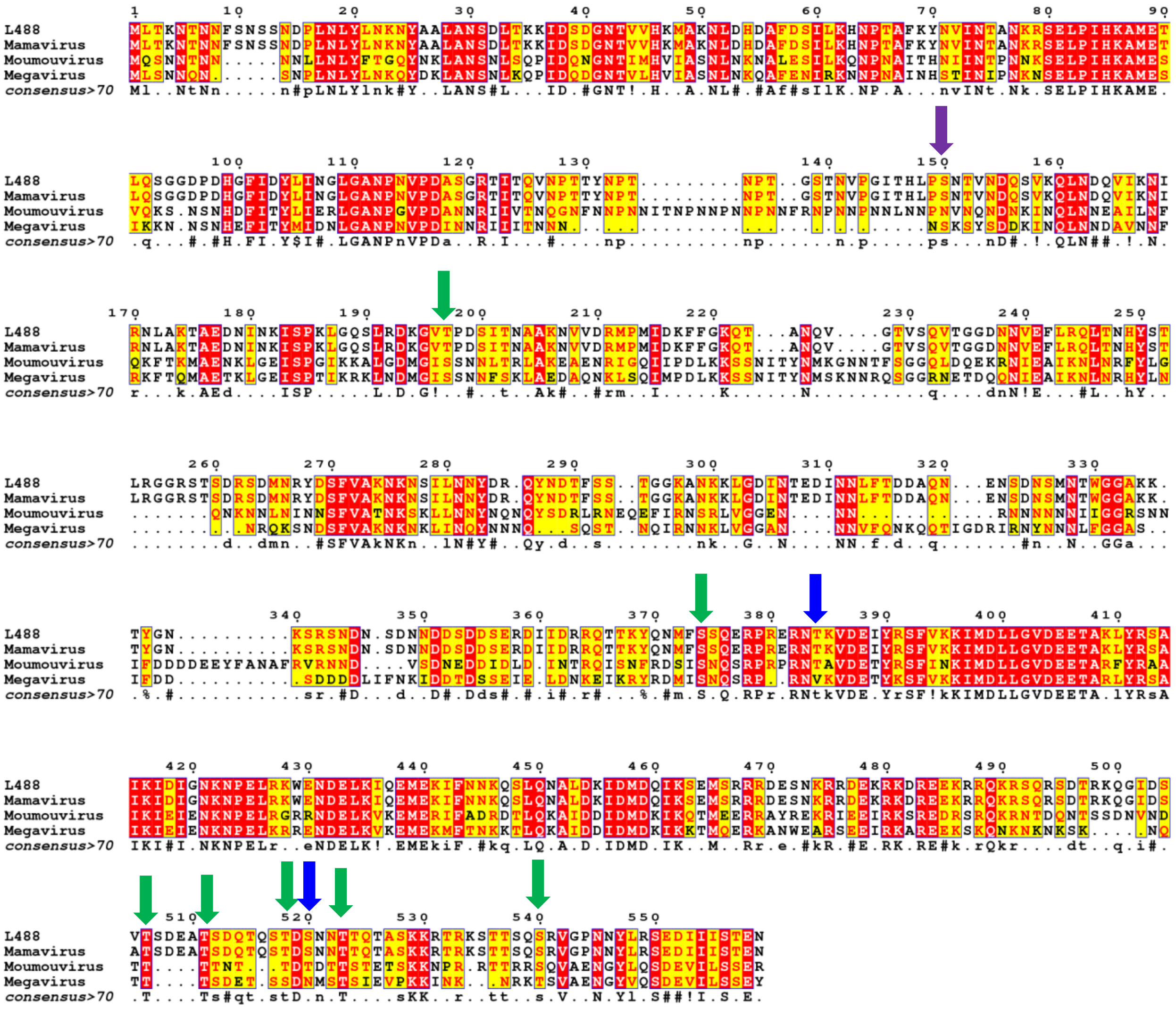

**Figure S8**. Structure-based alignment of L488 (YP_003986999) with its orthologs in the A clade (Mamavirus- AEQ60679), B clade (Moumouvirus-YP_007354295) and C clade (Megavirus chilensis-YP_00489443). The green arrow points to conserved residues potentially involved in O-glycosylation. The blue arrows indicate potentially O-glycosylation sites preserved only in the A and B clades. The violet arrow indicates potentially O-glycosylation sites preserved only in the A and C clades.

**Table S1**. NMR chemical shifts of poly_1 and poly_2. (600 MHz, 329 K, D2O) $^{1}H$ (plain) and $^{13}C$ (italic) chemical shifts of the two polysaccharides associated to Mimivirus fibrils. The two glucosamines (residues **B** and **D**) are *N*-acetylated ($^{1}H$ 2.09, $^{13}C$ 23.8). Viosamine is acetylated to the 4-amino function ($^{1}H$ 2.07, $^{13}C$ 23.5) and methylated at O-2 (methyl group signals at $^{1}H/^{13}C$ 3.63/60.8 ppm).

| | | 1 | 2 | 3 | 4 | 5 | 6 |
|---|---|---|---|---|---|---|---|
| Poly_1 | **C** | 4.80 | 3.95 | 3.87 | 3.42 | 4.29 | 1.24 |
| | **3-α-Rha** | *101.9* | *71.4* | *82.2* | *72.1* | *69.8* | *18.0* |
| | **D** | 4.79 | 3.90 | 3.75 | 3.60 | 3.48 | 3.71/4.10 |
| | **3,4,6-β-GlcNAc** | *104.1* | *57.0* | *79.0* | *75.8* | *67.0* | *65.2* |
| | ***S*-Pyr** | - | - | 1.47 | | | |
| | **Pyruvic acid** | *176.1* | *102.7* | *26.2* | | | |
| Poly_2 | **A** | 5.02 | 4.14 | 3.87 | 3.41 | 3.98 | 1.24 |
| | **2,3-α-Rha** | *101.0* | *78.9* | *82.2* | *72.0* | *70.6* | *18.4* |
| | **A'** | 4.99 | 4.14 | 3.90 | 3.45 | 4.00 | 1.24 |
| | **2,3-α-Rha** | *101.0* | *78.9* | *82.2* | *72.0* | *70.6* | *18.4* |
| | **B** | 4.88 | 3.83 | 3.62 | 3.59 | 3.37 | 3.88; 3.84 |
| | **3-β-GlcNAc** | *102.5* | *56.6* | *82.8* | *69.5* | *76.9* | *61.7* |
| | **E** | 4.59 | 3.22 | 3.56 | 3.64 | 3.54 | 1.24 |
| | **β-2OMeVio4NAc** | *105.3* | *84.3* | *73.9* | *57.8* | *72.0* | *18.3* |
| | **E'** | 4.58 | 3.46 | 3.55 | 3.63 | 3.59 | 1.23 |
| | **β-VioNAc** | *105.4* | *75.0* | *74.5* | *57.6* | *71.9* | *18.4* |

**Table S2**. Conservation level of the 29 proteins most enriched in the fibrils in the Acanthamoeba infecting mimiviruses. Values are based on a tBlastn search over the genomes available for all the viruses of the three clades of the *Mimiviridae* family. For all the proteins of Mimivirus, the PTM column indicates if any post-translation modification was detected by Renesto et al.[15] The 3 underlined are described as occurring in the fibrils by Sobhy et al.[16] The text in gray denotes the 7 proteins that were poorly conserved between the clades. In bold are the best candidates to act as carrier of the polysaccharides (this work). Proteins are sorted according to the ratings calculated from the first proteomic data set (SourceData File_1). The full list of the genome sequences used is in Table S3.

| | PTM* | Conservation Level (%) | | | | | | | |
|---|---|---|---|---|---|---|---|---|---|
| | | Clade A | | Clade B | | Clade C | | M4 | |
| Protein | | Query cover | Identity | Query cover | Identity | Query cover | Identity | Query cover | Identity |
| R135 | Y | 100 | 95 | 92 | 67 | 88 | 68 | - | - |
| L724 | Y | 100 | 82 | 69 | 67-68 | 69 | 69 | 100 | 82 |
| L567 | Y | 85 | 94 | 84 | 45 | 84 | 47-48 | 100 | 85 |
| R362 | N | 100 | 96 | 31 | 42 | 100 | 34 | 100 | 96 |
| L829 | Y | 100 | 100 | 93 | 60 | 97 | 60 | - | - |
| L690 | Y | 100 | 100 | 97 | 75 | 97 | 73 | 100 | 100 |
| L719 | ND | 100 | 84 | 48 | 38 | 50 | 38 | 100 | 84 |
| R387 | N | 100 | 77 | 81 | 34 | 98 | 30 | 100 | 77 |
| R610 | ND | 100 | 63 | 33-44 | 66 | 33 | 66 | 100 | 63 |
| R648 | N | 100 | 95 | 90 | 70-75 | 90 | 77 | 100 | 95 |
| L688 | Y | 100 | 89 | 99 | 47 | 99 | 50 | 100 | 89 |
| L485 | N | 100 | 100 | 87 | 63 | 89 | 62-63 | 100 | 100 |
| L894/L893 | NP | 100 | 96 | 89 | 63 | 89 | 62 | - | - |
| R346 | ND | 100 | 82 | - | - | - | - | 100 | 82 |
| R553 | Y | 97 | 98 | 79 | 40 | 85 | 38 | 97 | 98 |
| L488 | NP | 100 | 75 | 64-77 | 35 | 77 | 39 | 100 | 75 |
| L330 | N | 100 | 87 | 66 | 77 | 73 | 69 | 100 | 87 |
| R653 | N | 100 | 100 | 97 | 56 | 99 | 56 | 100 | 100 |
| R459 | Y | 100 | 88 | 73 | 76 | 72 | 78 | 100 | 88 |
| R188 | N | 100 | 100 | 95 | 50 | 98 | 48 | 100 | 100 |
| L725 | Y | 100 | 100 | 99 | 55 | 95 | 56 | 100 | 100 |
| L778 | NP | 92 | 100 | 81 | 48-52 | 91 | 43-45 | 91 | 100 |
| L399 | NP | 73 | 78 | 37 | 40 | 38 | 42 | 73 | 78 |
| R489 | Y | 83 | 93 | 70 | 52 | 70 | 50 | 83 | 93 |
| R727 | ND | 63 | 83 | 35 | 50 | 33 | 52 | 63 | 83 |
| L612 | N | 100 | 91 | 78-82 | 54-62 | 76 | 60 | 100 | 91 |
| L236 | ND | 100 | 100 | 93 | 55 | 88 | 56 | 100 | 100 |
| R710 | NP | 100 | 96 | 88 | 30 | 88-89 | 30 | 100 | 96 |
| L645 | ND | 100 | 54 | 14 | 51 | 11 | 65 | 100 | 54 |

*post-translation modification as reported from Renesto et al.; Y = yes, N = No, NP = not positioned in the 2D gel electrophoresis analysis; ND = not detected.

**Table S3**. Genomes accession numbers used to derive the data in Table S2. NCBI accession number of the complete genome sequences used in the tBlastn search to determine the conservation level (Supporting Table 2) of the proteins in the fibrils in the Acanthamoeba infecting Mimiviruses.

| Source | Access number | Clade |
|---|---|---|
| **Mimivirus** | AY653733 | A |
| **Mimivirus_batlle57** | CWJZ00000000 | A |
| **Mimivirus_battle19** | CWJX00000000 | A |
| **Mimivirus_battle27** | CWJS00000000 | A |
| **Mimivirus_battle66** | CWKA00000000 | A |
| **Mimivirus_battle6** | CWJU00000000 | A |
| **Mimivirus_battle7** | CWJW00000000 | A |
| **Mimivirus_battle83** | CWKG00000000 | A |
| **Mimivirus_battle86.fa** | CWKD00000000 | A |
| **Mimivirus_Bombay.fa** | KU761889 | A |
| **Mimivirus_dakar4** | CWKF00000000 | A |
| **Mimivirus_fauteuil** | LN871163.1 | A |
| **Mimivirus_kasaii** | AP017644.1 | A |
| **Mimivirus_Kroon** | KM982402 | A |
| **Mimivirus_lactour** | CXOL00000000 | A |
| **Mimivirus_M4** | JN036606 | A |
| **Mimivirus_Oyster** | KM982401 | A |
| **Mimivirus_pointerouge1** | LN871174 | A |
| **Mimivirus_pointerouge2** | LN871172.1 | A |
| **Mimivirus_shirakomae** | AP017645 | A |
| **Mimivirus_T2** | CXOT00000000 | A |
| **Mimivirus_T3** | CXOR00000000 | A |
| **Mimivirus_terra2** | NC_023639. | A |
| **Niemeyer_virus** | KT599914.1 | A |
| **Samba_virus** | KF959826.2 | A |
| **Lentillevirus** | AFYC01000005 | A |
| **Hirudovirus_strain_Sangsue** | KF493731.1 | A |
| **Mamavirus** | JF801956 | A |
| **Moumouvirus_battle49** | LN871171. | B |
| **Moumouvirus australensis** | MG807320 | B |
| **Moumouvirus** | MG807320 | B |
| **Moumouvirus_goulette** | KC008572. | B |
| **Moumouvirus_saoudian** | CXOQ00000000 | B |
| **Moumouvirus_saudi** | KY110734 | B |
| **Megavirus_battle43** | LN868256. | C |
| **Megavirus_avenue9** | LN867403.1 | C |
| **Megavirus_bus** | LN868539 | C |
| **Megavirus_curdo7** | ASM296631v1 | C |
| **Megavirus_chiliensis** | NC_016072. | C |
| **Megavirus_courdo11** | JX975216 | C |
| **Megavirus_J3** | CWIN00000000 | C |
| **Megavirus_lba** | JX885207 | C |
| **Megavirus_mont1** | CWIO00000000 | C |
| **Megavirus_montpellier3** | LN868518.1 | C |
| **Megavirus_Powai_lake** | KU877344. | C |
| **Megavirus_shan** | LN868520 | C |
| **Megavirus_T1** | LN868526 | C |
| **Megavirus_T4** | LN869537 | C |
| **Megavirus_T6** | CWJQ00000000 | C |
| **Megavirus_terra** | KF527229 | C |
| **Megavirus_ursino.fa** | CWJY00000000 | C |

**References**

[1] D. Byrne, R. Grzela, A. Lartigue, S. Audic, S. Chenivesse, S. Encinas, J.-M. Claverie, C. Abergel, *Genome Res.* **2009**, *19*, 1233-1242.

[2] C. Xiao, Y. G. Kuznetsov, S. Sun, S. L. Hafenstein, V. A. Kostyuchenko, P. R. Chipman, M. Suzan-Monti, D. Raoult, A. McPherson, M. G. Rossmann, *PLoS Biology* **2009**, *7*, e1000092.

[3] C. De Castro, M. Parrilli, O. Holst, A. Molinaro, in *Methods Enzymol.* **2010***, 480,*. 89-115.

[4] J. Lönngren, S. Svensson, in *Adv. Carbohydr. Chem. Biochem.* **1974***, 29,*. 41-106.

[5] F. Piacente, C. De Castro, S. Jeudy, M. Gaglianone, M. E. Laugieri, A. Notaro, A. Salis, G. Damonte, C. Abergel, M. G. Tonetti, *J. Biol. Chem.* **2017**, *292*, 7385-7394.

[6] M. Parakkottil Chothi, G. A. Duncan, A. Armirotti, C. Abergel, J. R. Gurnon, J. L. Van Etten, C. Bernardi, G. Damonte, M. Tonetti, *J. Virol.* **2010**, *84*, 8829-8838.

[7] F. Piacente, C. Bernardi, M. Marin, G. Blanc, C. Abergel, M. G. Tonetti, *Glycobiology* **2014**, *24*, 51-61.

[8] F. Piacente, M. Marin, A. Molinaro, C. De Castro, V. Seltzer, A. Salis, G. Damonte, C. Bernardi, J.-M. Claverie, C. Abergel, M. Tonetti, *J. Biol. Chem.* **2012**, *287*, 3009-3018.

[9] Hager, Sützl, Stefanović, Blaukopf, Schäffer, *Int. J. Mol. Sci.* **2019**, *20*, 4929.

[10] A. Salvetti, Y. Couté, A. Epstein, L. Arata, A. Kraut, V. Navratil, P. Bouvet, A. Greco, *J. Proteome Res.* **2016**, *15*, 1659-1669.

[11] D. Bouyssié, A.-M. Hesse, E. Mouton-Barbosa, M. Rompais, C. Macron, C. Carapito, A. Gonzalez de Peredo, Y. Couté, V. Dupierris, A. Burel, J.-P. Menetrey, A. Kalaitzakis, J. Poisat, A. Romdhani, O. Burlet-Schiltz, S. Cianférani, J. Garin, C. Bruley, *Bioinformatics* **2020**, *36*, 3148-3155.

[12] J.-M. Claverie, T. N. Ta, *Bioinformatics* **2018**, *35*, 170-171.

[13] F. Armougom, S. Moretti, O. Poirot, S. Audic, P. Dumas, B. Schaeli, V. Keduas, C. Notredame, *Nucleic Acids Res.* **2006**, *34*, W604-608.

[14] P. Gouet, X. Robert, E. Courcelle, *Nucleic Acids Res.* **2003**, *31*, 3320-3323.

[15] P. Renesto, C. Abergel, P. Decloquement, D. Moinier, S. Azza, H. Ogata, P. Fourquet, J.-P. Gorvel, J.-M. Claverie, *J. Virol.* **2006**, *80*, 11678-11685.

[16] H. Sobhy, B. L. Scola, I. Pagnier, D. Raoult, P. Colson, *Front. Microbiol.* **2015**, *6*.

## Author Contributions

A. N. purified the fibrils, analyzed the NMR and the proteomic data, performed SDS-PAGE and HPLC analyses, drafted the manuscript, A. M. performed NMR experiments, analyzed the data and contributed to write the manuscript, Y. C. designed proteomic experiments on the whole and defibrillated virus, and on the fibers, analyzed all the proteomic data and contributed to write the manuscript, L. B. performed proteomic experiments, M. E. L. analyzed the viral chemical composition, A. S., G. D. and M. T. performed the proteomic experiments, M. T., C. A. and C. D. C designed the study, analyzed results, and wrote the manuscript.
